# Supplementary material for: Transcriptomic and anatomic parcellation of 5-HT3AR expressing cortical interneuron subtypes revealed by single-cell RNA sequencing
Source: Nat Commun. 2017 Jan 30;8:14219. doi: 10.1038/ncomms14219 (PMC5290279; doi:10.1038/ncomms14219)
Supplement: Supplementary Information — Supplementary Figures and Supplementary Tables. [file ncomms14219-s1.pdf]

Supplementary Figure 1

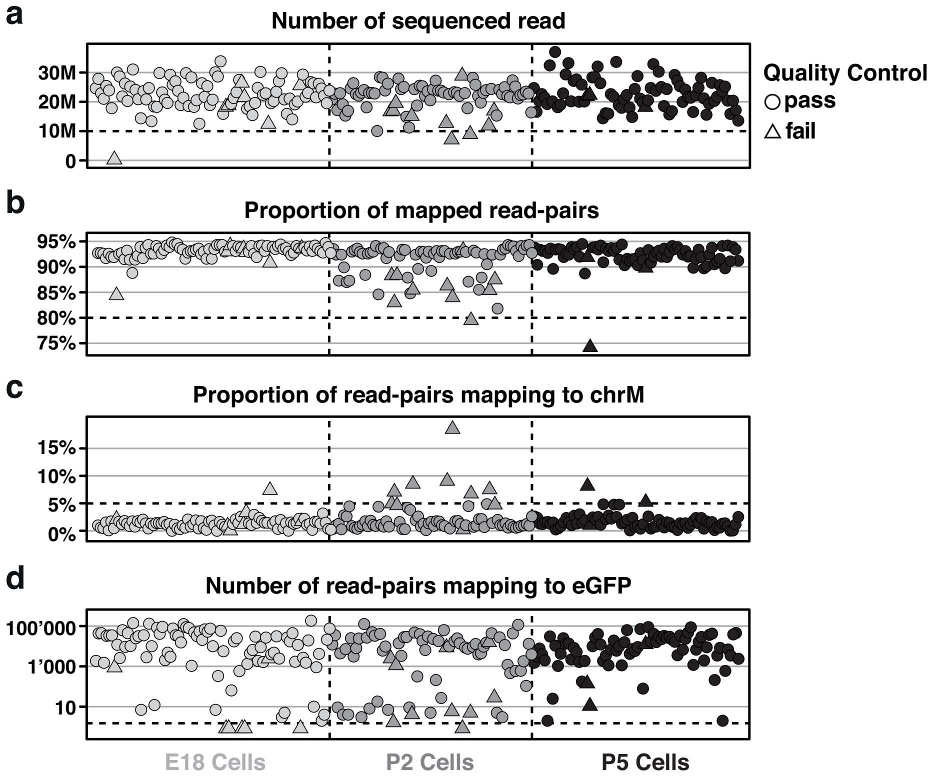

Supplementary Figure 1. Quality control (QC) steps for the selection of *Htr3a*-GFP+ cells. Single cell RNA-seq was performed on a total of 243 single cells obtained using microfluidic chips at E18, P2 and P5 (n = 89 cells at E18 in 2 chips; n = 76 cells at P2 in 1 chip; n = 78 cells at P5 in 2 chips). a) Cells passed QC if at least 5 million read-pairs were sequenced per cell. b) Cells passed QC if more than 80% of the read-pairs were mapped to the genome. c) Cells passed QC if less than 5% of the reads were mapped to the mitochondrial chromosome (chrM). d) Cells passed QC if at least one read was aligned to eGFP. A total of 223 cells (E18: 82 cells; P2: 66 cells; P5: 75 cells) satisfied all 4 QC criterions.

Supplementary Figure 2

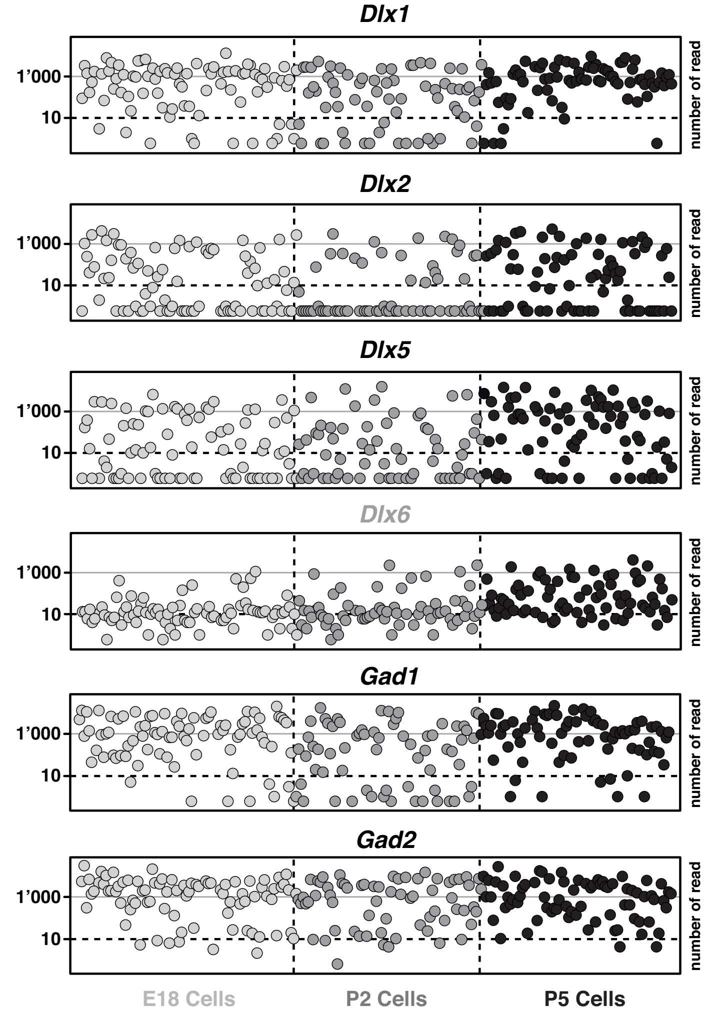

Supplementary Figure 2. *Htr3a*-GFP+ cells selected for data analysis express interneuron markers. All 223 cells that passed quality control had at least ten sequence reads aligned to at least one of the following interneuron-specific markers: *Dlx1*, *Dlx2*, *Dlx5*, *Dlx6, Gad1*, *Gad2.*

Supplementary Figure 3

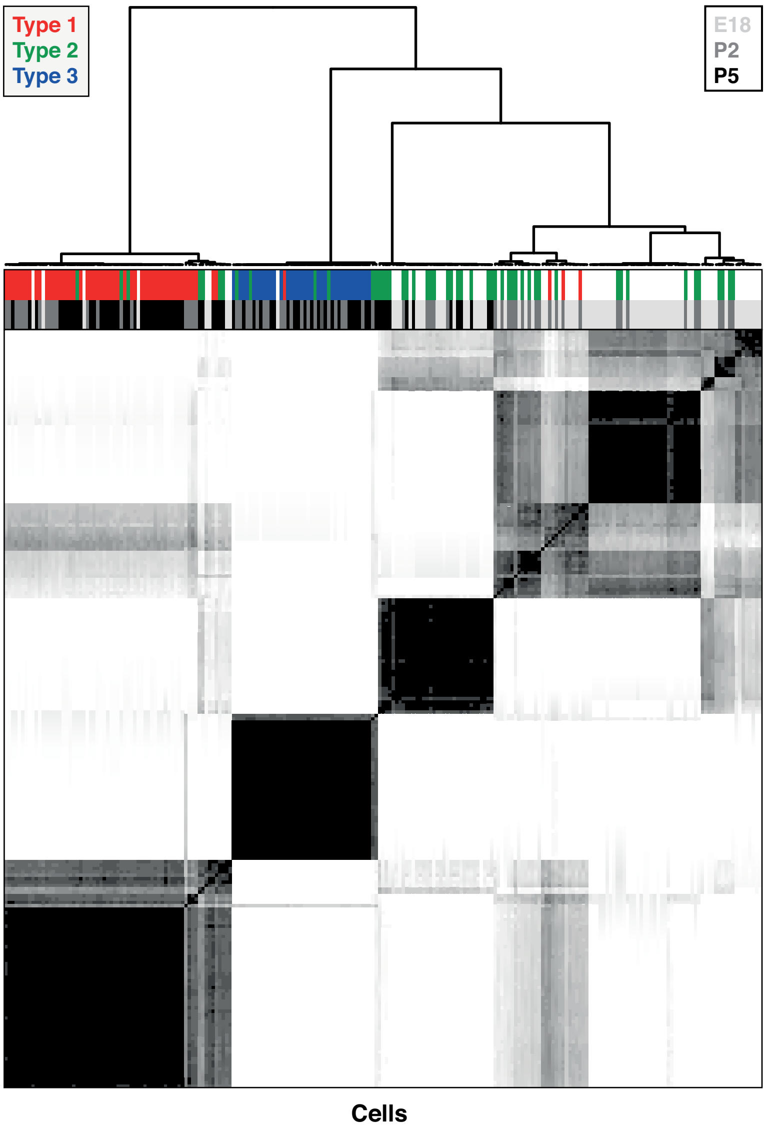

Supplementary Figure 3. Cluster stability analysis using random sampling combined with principal component analysis was performed on cells at E18, P2 and P5. Hierarchical clustering revealed that *Htr3a*-GFP+ INs at P2 and P5 clustered primarily based on type-identity (color-coded) and secondarily based on developmental age (grey coding).

Supplementary Figure 4

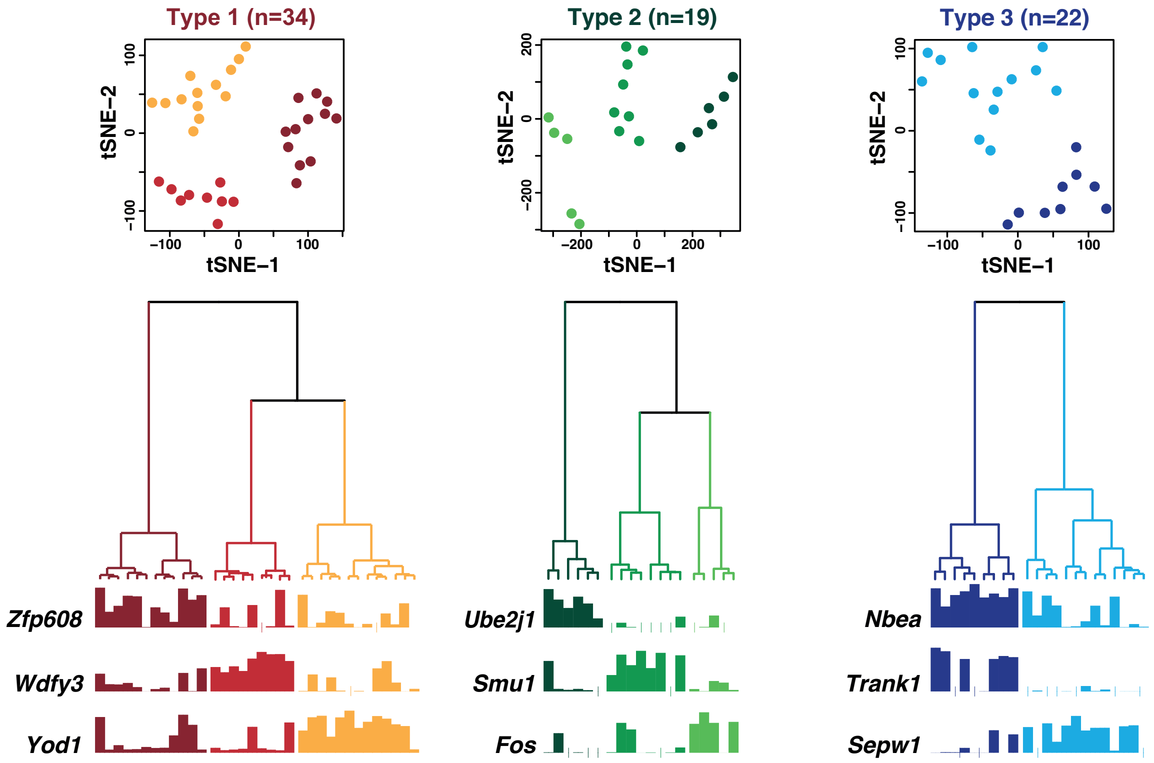

Supplementary Figure 4. t-Distributed Stochastic Neighbor Embedding (t-SNE) [43] analysis and hierarchical clustering reveals that type 1, type 2, and type 3 *Htr3a*-GFP+ INs comprise several molecularly distinct subtypes of *Htr3a*-GFP+ INs at P5. Illustrative subtype marker genes are displayed.

Supplementary Figure 5

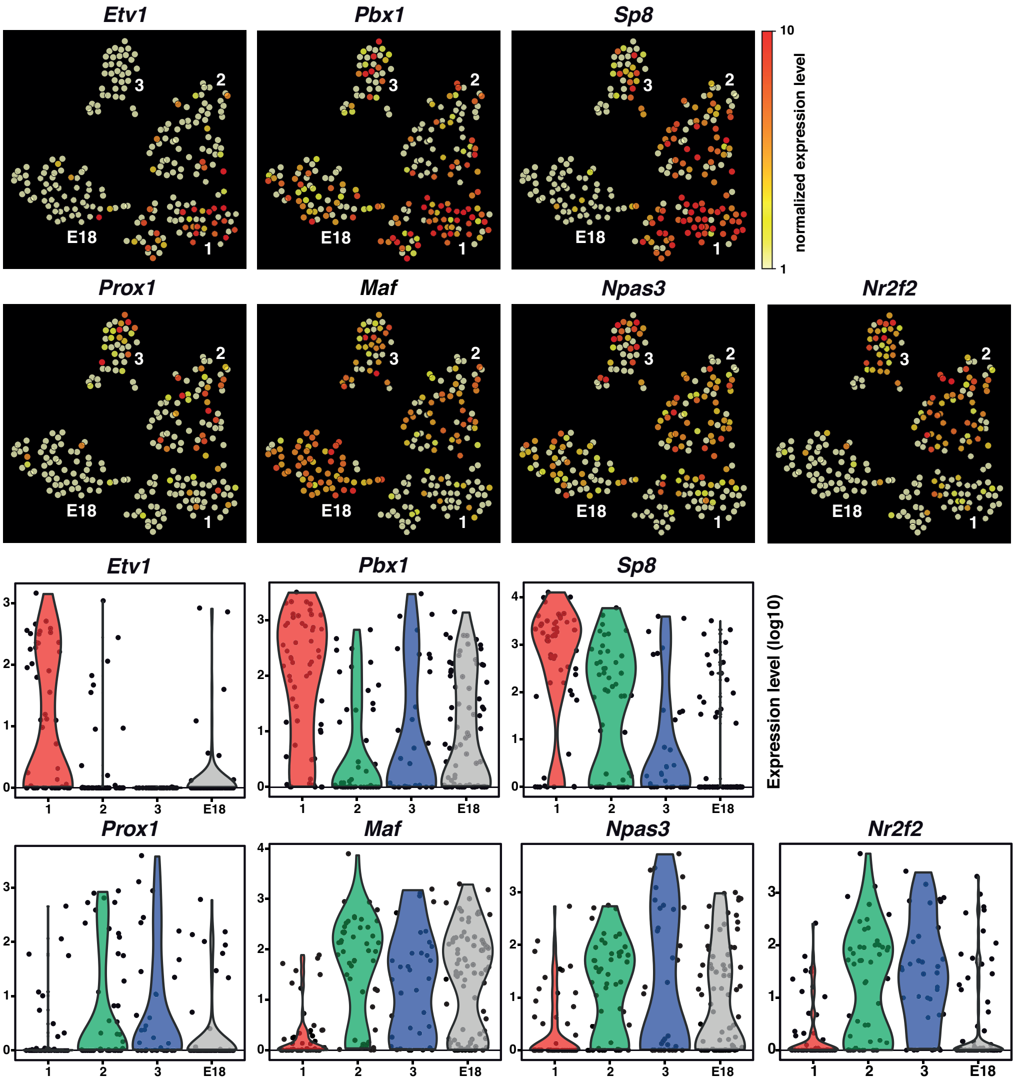

Supplementary Figure 5. Transcription factors (TFs) *Etv1, Pbx1, Sp8* expressed in olfactory bulb INs are enriched in type 1 INs whereas CGE-expressed TFs *Prox1, Maf*, *Npas3*, *Nr2f2* are enriched in type 2 and 3 INs. a) t-Distributed Stochastic Neighbor Embedding (t-SNE) analysis indicates that *Etv1, Pbx1, Sp8* are enriched in type 1 INs whereas *Prox1, Maf*, *Npas3*, *Nr2f2* are enriched in types 2 and 3 INs. Graph plots and gene expression levels were obtained using the Seurat R package ouput [43] b) Violin plots showing increased expression of *Etv1, Pbx1, Sp8* in type 1 INs and increased expression of *Prox1, Maf*, *Npas3*, *Nr2f2* in types 2 and 3 INs. Gene expression levels were obtained using the join posterior estimation from the SCDE R package [44].

Supplementary Figure 6

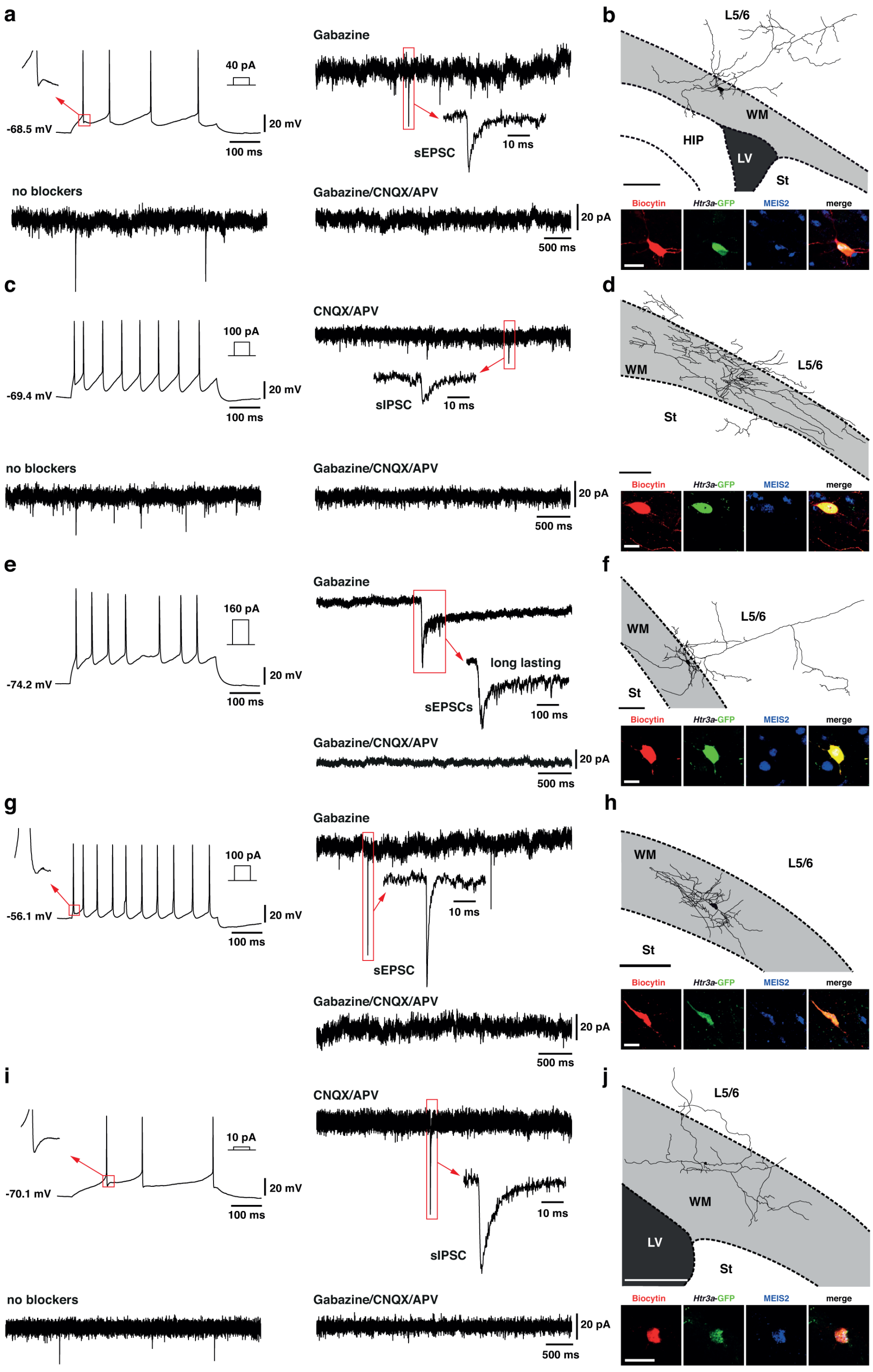

Supplementary Figure 6. MEIS2+ / *Htr3a*-GFP+ interneurons (INs) are functionally integrated in the juvenile (P21-P28) cortical white matter (WM). a, c, e, g, i) Example traces of *Htr3a*-GFP+ cell recordings in current and voltage clamp mode. Cells responded to depolarizing current injections with a variety of spiking patterns (left top). Note the inserts showing magnifications of the multiphasic after-hyperpolarization potentials observed in the first spikes of some cells. Mixed sEPSCs and sIPSCs were recorded in the absence of blockers (left bottom in a, c and i). sEPSCs were recorded in the presence of Gabazine (right top in a,e and g) and were abolished upon addition of CNQX and APV (right bottom in a,e and g). Note the long-lasting excitation due to multiple sEPSCs observed in e. sIPSCs were recorded in the presence of CNQX and APV (right top in c and i) and were abolished upon addition of Gabazine (right bottom in c and i). b, d, f, h, j) Reconstruction of WM *Htr3a*-GFP+ recorded cells expressing MEIS2 and extending processes in the WM, overlying cortical layers 5/6 and hippocampus (HIP, in b) or striatum (St, in d). LV: lateral ventricle. Graphs display mean ± SEM. Scale bars: (b, d, f, h, j) 100 µm for reconstructions and 20 µm for high magnification images.

Supplementary Figure 7

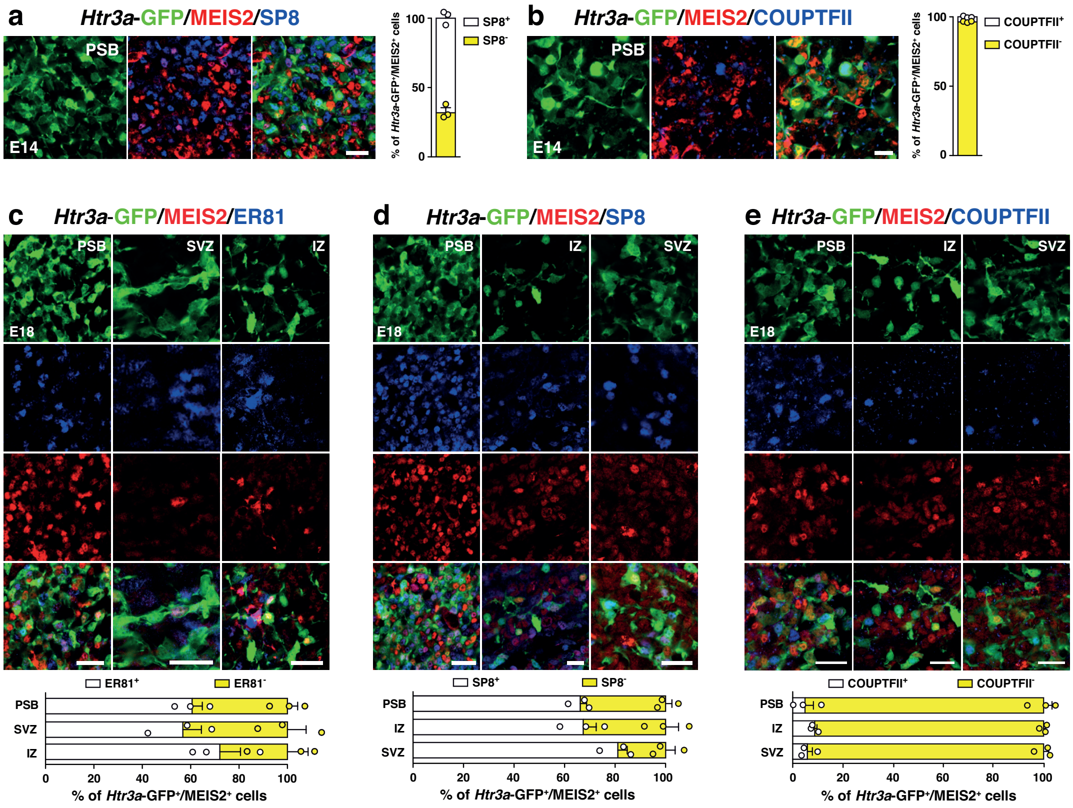

Supplementary Figure 7. *Htr3a*-GFP+ INs located at the embryonic pallial-subpallial boundary (PSB) and in the pallium express SP8 and ER81 but more rarely COUPTFII. a) At E14, MEIS2+ /*Htr3a*-GFP+ INs located at the PSB express SP8 b) At E14, MEIS2+ /*Htr3a*-GFP+ INs located at the PSB more rarely express COUPTFII. c) At E18, a large fraction of MEIS2+ / *Htr3a*-GFP+ INs at the PSB, in the tangential migratory stream and in the IZ express ER81. d) At E18, a large fraction of MEIS2+ / *Htr3a*-GFP+ INs at the PSB, in the tangential migratory stream and in the IZ express SP8. e) At E18, MEIS2+ / *Htr3a*-GFP+ INs at the PSB, in the tangential migratory stream and in the IZ more rarely express COUPTFII. Graphs display mean ± S.E.M and n = 3 brains for each. Scale bars: (a-e) 20µm.

Supplementary Figure 8

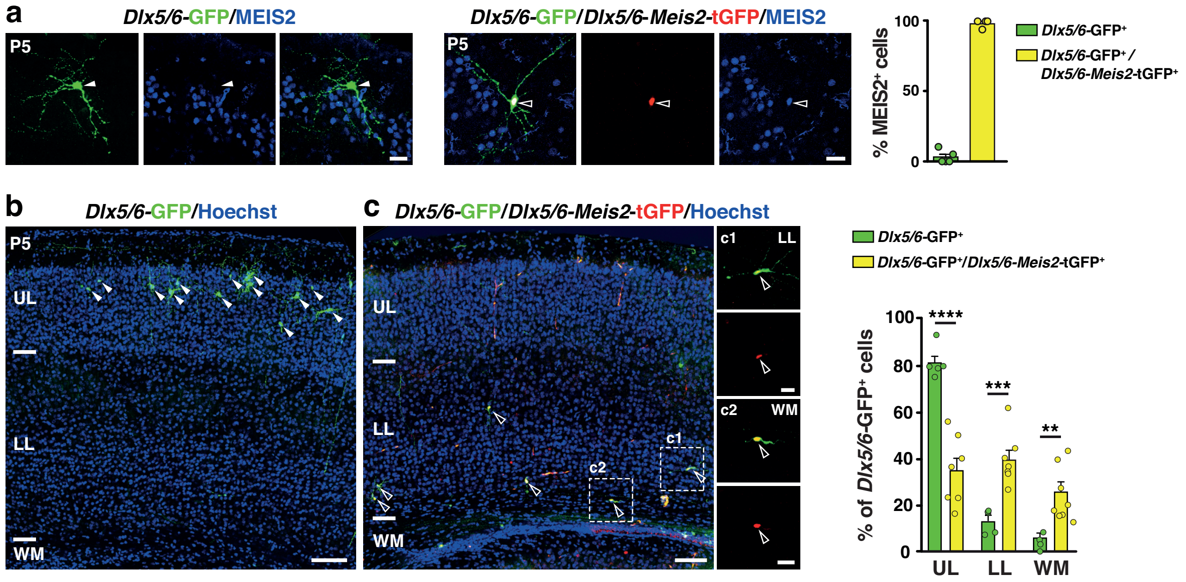

Supplementary figure 8: Ectopic expression of MEIS2 in CGE-derived *Htr3a*-GFP+ interneurons (INs) shifts their laminar positioning to the white matter (WM) and lower cortical layers. a) *In utero* electroporation targeting the E14 CGE was used to ectopically express MEIS2 in CGE-derived INs. At P5, control *Dlx5/6*-GFP+ electroporated INs located in the cortex did not express MEIS2 (arrowheads; n = 5 brains, 354 cells) in contrast to double-labeled *Dlx5/6*-GFP+ / *Dlx5/6-Meis2*-tGFP+ INs (open arrowheads; n = 4 brains, 98 cells). b) At P5, *Dlx5/6*-GFP+ / *Dlx5/6-Meis2*-tGFP+ significantly shifted their laminar location to the white matter (WM) and lower cortical layers (LL) (open arrowheads; n = 7 brains, 199 cells) compared to control *Dlx5/6*-GFP+ INs (arrowheads; n = 5 brains, 354 cells), which were preferentially located in upper cortical layers (UL) (**** $P < 0.001$, *** $P < 0.005$, ** $P < 0.01$, student *t*-test). Scale bars: (b, c) 100 µm for low magnification images; (a, c) 20 µm for high magnification images.

Supplementary Figure 9

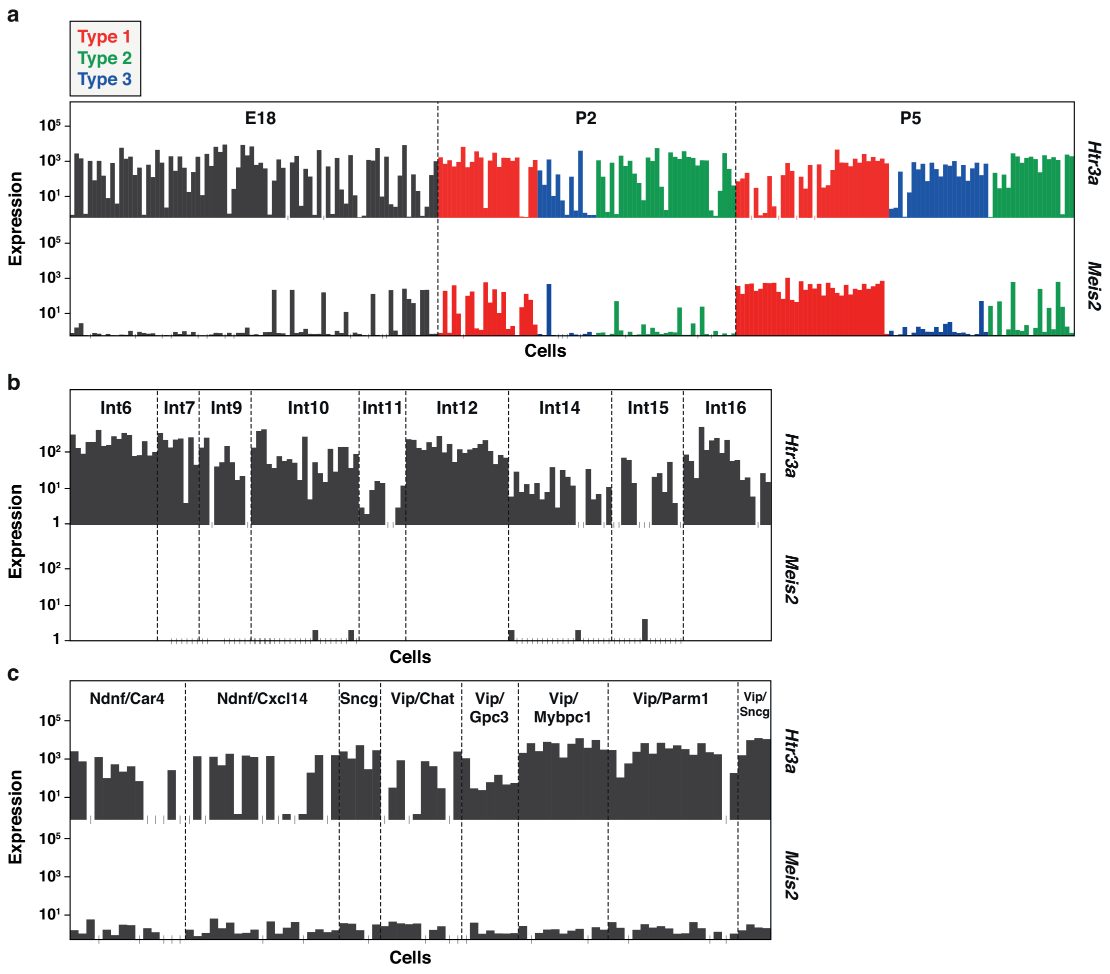

Supplementary Figure 9. a) Expression of the *Meis2* transcript was enriched in type 1 but not in type 2 or type 3 *Htr3a*-GFP+ INs. b, c) Expression of the *Meis2* transcript was not enriched in *Htr3a*-expressing cortical INs isolated from the grey matter of the somatosensory cortex [35] (b) or visual cortex [34] (c).

Supplementary Figure 10

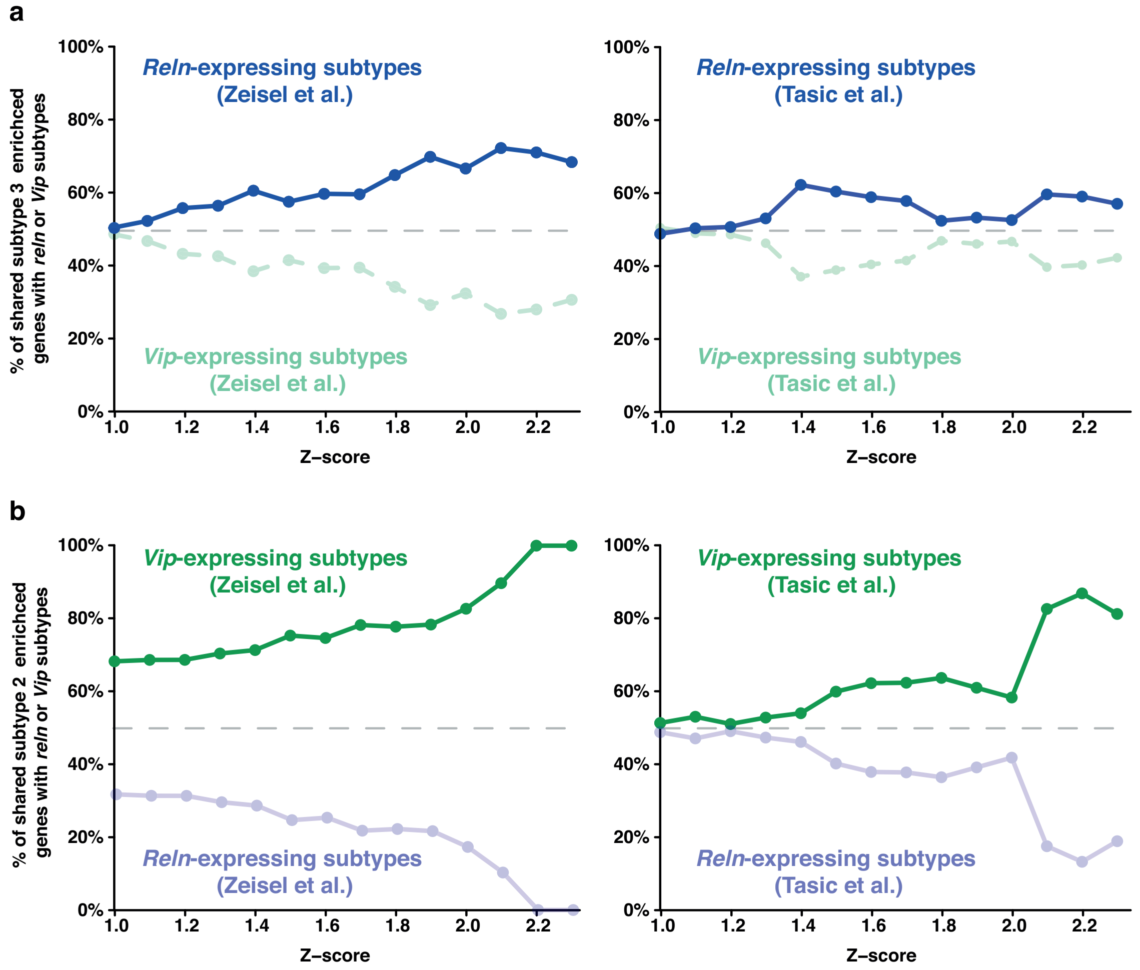

Supplementary Figure 10. Type 3 *Htr3a*-GFP+ INs showed higher transcriptional proximity with cortical INs belonging to *Reln*-enriched subtypes as compared to *Vip*-enriched subtypes identified in the gray matter of the somatosensory cortex [35] or visual cortex [34]. a) Type 3 *Htr3a*-GFP+ INs share increased percentage of enriched genes with *Reln*-enriched interneuron subtypes as compared to *Vip*-enriched interneuron subtypes identified in the gray matter by Zeisel et al [35] and Tasic et al [34] b) Type 2 *Htr3a*-GFP+ INs share increased percentage of enriched genes with *Vip*-enriched INs as compared to *Reln*-enriched INs identified in the gray matter by Zeisel et al [35] and Tasic et al [34]. Z-scores for enriched genes were determined using SCDE.

Supplementary Table 1. Genes up-regulated in type 1 *Htr3a*-GFP+ interneurons at P2

N°	Ensembl Identifier	Symbol	Description	Z-score	Fold Change (MLE)
1	ENSMUSG00000023800	*Tiam2*	T cell lymphoma invasion and metastasis 2	4.1	5.2
2	ENSMUSG00000034614	*Pik3ip1*	phosphoinositide-3-kinase interacting protein 1	3.7	6.5
3	ENSMUSG00000049811	*Fam161a*	family with sequence similarity 161, member A	3.5	6.3
4	ENSMUSG00000050558	*Prokr2*	prokineticin receptor 2	3.4	7.8
5	ENSMUSG00000085936	*2610307P16Rik*	RIKEN cDNA 2610307P16 gene	3.3	7.6
6	ENSMUSG00000038718	*Pbx3*	pre B cell leukemia homeobox 3	3.3	5.5
7	ENSMUSG00000029563	*Foxp2*	forkhead box P2	3.3	6.2
8	ENSMUSG00000027210	*Meis2*	Meis homeobox 2	3.1	4.2
9	ENSMUSG00000053841	*Txlna*	taxilin alpha	3.1	6.9
10	ENSMUSG00000104200	*RP23-172P6.1*	predicted gene, 37399	3.1	5.5
11	ENSMUSG00000028629	*Exo5*	exonuclease 5	3.1	6.5
12	ENSMUSG00000052534	*Pbx1*	pre B cell leukemia homeobox 1	3.0	3.5
13	ENSMUSG00000026766	*Mmadhc*	methylmalonic aciduria (cobalamin deficiency) cblD type, with homocystinuria	3.0	4.1
14	ENSMUSG00000042063	*Zfp386*	zinc finger protein 386 (Kruppel-like)	2.9	5.3
15	ENSMUSG00000000184	*Ccnd2*	cyclin D2	2.9	4.2
16	ENSMUSG00000053560	*Ier2*	immediate early response 2	2.8	4.7
17	ENSMUSG00000057388	*Mrpl18*	mitochondrial ribosomal protein L18	2.8	3.6
18	ENSMUSG00000102808	*RP23-380F8.1*	RIKEN cDNA 5430420F09 gene	2.8	5.1
19	ENSMUSG00000004151	*Etv1*	ets variant 1	2.7	4.0
20	ENSMUSG00000052684	*Jun*	jun proto-oncogene	2.7	3.0
21	ENSMUSG00000025408	*Ddit3*	DNA-damage inducible transcript 3	2.7	4.9
22	ENSMUSG00000005057	*Sh2b2*	SH2B adaptor protein 2	2.7	5.5
23	ENSMUSG00000086308	*G630016G05Rik*	RIKEN cDNA G630016G05 gene	2.7	5.5
24	ENSMUSG00000020160	*Meis1*	Meis homeobox 1	2.7	4.3
25	ENSMUSG00000023232	*Serinc2*	serine incorporator 2	2.7	5.9
26	ENSMUSG00000022489	*Pde1b*	phosphodiesterase 1B, Ca2+-calmodulin dependent	2.7	5.6
27	ENSMUSG00000038156	*Spon1*	spondin 1, (f-spondin) extracellular matrix protein	2.6	4.5
28	ENSMUSG00000038250	*Usp38*	ubiquitin specific peptidase 38	2.6	4.5
29	ENSMUSG00000036737	*Oxsr1*	oxidative-stress responsive 1	2.6	3.8
30	ENSMUSG00000043668	*Tox3*	TOX high mobility group box family member 3	2.6	5.2
31	ENSMUSG00000039194	*Rlbp1*	retinaldehyde binding protein 1	2.5	4.7
32	ENSMUSG00000028480	*Glipr2*	GLI pathogenesis-related 2	2.5	6.1
33	ENSMUSG00000061118	*Dnajc30*	DnaJ (Hsp40) homolog, subfamily C, member 30	2.5	5.3
34	ENSMUSG00000001120	*Pcbp3*	poly(rC) binding protein 3	2.5	5.8
35	ENSMUSG00000100561	*Gm7516*	predicted gene 7516	2.5	5.4
36	ENSMUSG00000021681	*Aggf1*	angiogenic factor with G patch and FHA domains 1	2.5	4.1
37	ENSMUSG00000097263	*Gm26804*	predicted gene, 26804	2.4	4.8
38	ENSMUSG00000028689	*Ccdc163*	coiled-coil domain containing 163	2.4	6.3
39	ENSMUSG00000019464	*Ptger1*	prostaglandin E receptor 1 (subtype EP1)	2.4	5.6
40	ENSMUSG00000020115	*Tbk1*	TANK-binding kinase 1	2.4	4.4
41	ENSMUSG00000031512	*Tex29*	testis expressed 29	2.4	5.4
42	ENSMUSG00000060961	*Slc4a4*	solute carrier family 4 (anion exchanger), member 4	2.4	6.7
43	ENSMUSG00000055612	*Cdca7*	cell division cycle associated 7	2.4	4.0
44	ENSMUSG00000057561	*Eif1a*	eukaryotic translation initiation factor 1A	2.4	3.6
45	ENSMUSG00000035181	*Heatr5a*	HEAT repeat containing 5A	2.4	5.4
46	ENSMUSG00000040859	*Bsdc1*	BSD domain containing 1	2.4	3.2
47	ENSMUSG00000028894	*Inpp5b*	inositol polyphosphate-5-phosphatase B	2.4	6.1
48	ENSMUSG00000097156	*Gm3764*	predicted gene 3764	2.4	2.3
49	ENSMUSG00000087481	*Gm15608*	predicted gene 15608	2.3	5.0
50	ENSMUSG00000024565	*Sall3*	sal-like 3 (Drosophila)	2.3	6.6
51	ENSMUSG00000028684	*Urod*	uroporphyrinogen decarboxylase	2.3	6.3
52	ENSMUSG00000029326	*Enoph1*	enolase-phosphatase 1	2.3	4.2
53	ENSMUSG00000002635	*Pdcd2l*	programmed cell death 2-like	2.3	5.0
54	ENSMUSG00000031971	*Ccsap*	centriole, cilia and spindle associated protein	2.3	6.0
55	ENSMUSG00000029246	*Ppat*	phosphoribosyl pyrophosphate amidotransferase	2.3	6.9
56	ENSMUSG00000071477	*Zfp777*	zinc finger protein 777	2.3	4.7
57	ENSMUSG00000045877	*4933415A04Rik*	RIKEN cDNA 4933415A04 gene	2.3	5.6
58	ENSMUSG00000024019	*Cmtr1*	cap methyltransferase 1	2.3	6.8
59	ENSMUSG00000031595	*Pdgfrl*	platelet-derived growth factor receptor-like	2.3	4.5
51	ENSMUSG00000066189	*Cacng3*	calcium channel, voltage-dependent, gamma subunit 3	-5.9	-2.8
52	ENSMUSG00000024109	*Nrxn1*	neurexin I	-3.8	-2.8
53	ENSMUSG00000078578	*Ube2d3*	ubiquitin-conjugating enzyme E2D 3	-4.5	-2.8
54	ENSMUSG00000031782	*Coq9*	coenzyme Q9 homolog (yeast)	-5.9	-2.8
55	ENSMUSG00000030077	*Chl1*	cell adhesion molecule with homology to L1CAM	-3.3	-2.8

56	ENSMUSG00000029174	*Tbc1d1*	TBC1 domain family, member 1	-5.9	-2.8
57	ENSMUSG00000031532	*Saraf*	store-operated calcium entry-associated regulatory factor	-4.8	-2.8
58	ENSMUSG00000048661	*Lemd3*	LEM domain containing 3	-5.9	-2.8
59	ENSMUSG00000024560	*Cxxc1*	CXXC finger 1 (PHD domain)	-6.3	-2.7
60	ENSMUSG00000040022	*Rab11fip2*	RAB11 family interacting protein 2 (class I)	-5.9	-2.7
61	ENSMUSG00000026277	*Stk25*	serine/threonine kinase 25 (yeast)	-6.4	-2.7
62	ENSMUSG00000003411	*Rab3b*	RAB3B, member RAS oncogene family	-6.4	-2.7
63	ENSMUSG00000024897	*Apba1*	amyloid beta (A4) precursor protein binding, family A, member 1	-6.0	-2.7
64	ENSMUSG00000035236	*Scai*	suppressor of cancer cell invasion	-5.1	-2.7
65	ENSMUSG00000026696	*Vamp4*	vesicle-associated membrane protein 4	-5.6	-2.7
66	ENSMUSG00000085793	*Lin52*	lin-52 homolog (C. elegans)	-6.1	-2.7
67	ENSMUSG00000029608	*Rph3a*	rabphilin 3A	-4.2	-2.7
68	ENSMUSG00000025485	*Ric8*	resistance to inhibitors of cholinesterase 8 homolog (C. elegans)	-6.3	-2.7
69	ENSMUSG00000029063	*Nadk*	NAD kinase	-6.8	-2.7
70	ENSMUSG00000046447	*Camk2n1*	calcium/calmodulin-dependent protein kinase II inhibitor 1	-5.0	-2.7
71	ENSMUSG00000031841	*Cdh13*	cadherin 13	-6.1	-2.7
72	ENSMUSG00000055373	*Fut9*	fucosyltransferase 9	-6.0	-2.7
73	ENSMUSG00000001089	*Luzp1*	leucine zipper protein 1	-5.8	-2.7
74	ENSMUSG00000020817	*Rabep1*	rabaptin, RAB GTPase binding effector protein 1	-4.9	-2.7
75	ENSMUSG00000044224	*Dnajc21*	DnaJ (Hsp40) homolog, subfamily C, member 21	-6.6	-2.7
76	ENSMUSG00000033478	*Fam160b1*	family with sequence similarity 160, member B1	-6.0	-2.7
77	ENSMUSG00000040152	*Thbs1*	thrombospondin 1	-5.9	-2.7
78	ENSMUSG00000029038	*Ssu72*	Ssu72 RNA polymerase II CTD phosphatase homolog (yeast)	-5.5	-2.7
79	ENSMUSG00000024376	*Epb4.1l4a*	erythrocyte protein band 4.1-like 4a	-5.6	-2.7
80	ENSMUSG00000018428	*Akap1*	A kinase (PRKA) anchor protein 1	-6.1	-2.7
81	ENSMUSG00000042229	*Rabif*	RAB interacting factor	-6.6	-2.6
82	ENSMUSG00000032878	*Ccdc85a*	coiled-coil domain containing 85A	-5.8	-2.6
83	ENSMUSG00000041986	*Elmod1*	ELMO/CED-12 domain containing 1	-5.0	-2.6
84	ENSMUSG00000048490	*Nrip1*	nuclear receptor interacting protein 1	-6.1	-2.6
85	ENSMUSG00000019970	*Sgk1*	serum/glucocorticoid regulated kinase 1	-5.3	-2.6
86	ENSMUSG00000084390	*Gm15425*	predicted gene 15425	-5.8	-2.6
87	ENSMUSG00000031530	*Dusp4*	dual specificity phosphatase 4	-5.6	-2.6
88	ENSMUSG00000060098	*Prmt7*	protein arginine N-methyltransferase 7	-5.8	-2.6
89	ENSMUSG00000060988	*Galnt13*	UDP-N-acetyl-alpha-D-galactosamine:polypeptide N-acetylgalactosaminyltransferase 13	-5.8	-2.6
90	ENSMUSG00000028528	*Dnajc6*	DnaJ (Hsp40) homolog, subfamily C, member 6	-5.9	-2.6
91	ENSMUSG00000030704	*Rab6a*	RAB6A, member RAS oncogene family	-4.2	-2.6
92	ENSMUSG00000060181	*Slc35e3*	solute carrier family 35, member E3	-5.6	-2.6
93	ENSMUSG00000049985	*Ankrd55*	ankyrin repeat domain 55	-5.7	-2.6
94	ENSMUSG00000070866	*Zfp804a*	zinc finger protein 804A	-5.8	-2.6
95	ENSMUSG00000026109	*Tmeff2*	transmembrane protein with EGF-like and two follistatin-like domains 2	-5.2	-2.6
96	ENSMUSG00000062906	*Hdac10*	histone deacetylase 10	-6.7	-2.6
97	ENSMUSG00000037747	*Phyhipl*	phytanoyl-CoA hydroxylase interacting protein-like	-6.1	-2.6
98	ENSMUSG00000030521	*Mphosph10*	M-phase phosphoprotein 10 (U3 small nucleolar ribonucleoprotein)	-5.3	-2.6
99	ENSMUSG00000039183	*Nubp2*	nucleotide binding protein 2	-6.0	-2.6
100	ENSMUSG00000008450	*Nutf2*	nuclear transport factor 2	-5.8	-2.6

Legend: List of genes displaying a significant up-regulation in type 1 *Htr3a*-GFP+ cortical interneurons (INs) compared to type 2 and 3 INs at P2. Genes are identified using single-cell differential display (SCDE) with a Maximum Likelihood Estimate (MLE) ≥ 2 and Z-score ≥ 2. Ranking of genes is based on Z-scores. Top 100 genes are displayed. Additional enriched genes can be found in the Supplementary data file. The genes highlighted in red are presented in figure 1.

Supplementary Table 2: Genes down-regulated in type 1 *Htr3a*-GFP+ interneurons at P2

N°	Ensembl Identifier	Symbol	Description	Z-score	Fold Change (MLE)
1	ENSMUSG00000033066	*Gas7*	growth arrest specific 7	-8.1	-4.2
2	ENSMUSG00000056222	*Spock1*	sparc/osteonectin, cwcv and kazal-like domains proteoglycan 1	-7.9	-3.9
3	ENSMUSG00000056515	*Rab31*	RAB31, member RAS oncogene family	-7.1	-3.7
4	ENSMUSG00000020053	*Igf1*	insulin-like growth factor 1	-7.9	-3.7
5	ENSMUSG00000035776	*Cd99l2*	CD99 antigen-like 2	-7.6	-3.7
6	ENSMUSG00000037119	*D15Ertd621e*	DNA segment, Chr 15, ERATO Doi 621, expressed	-7.5	-3.6
7	ENSMUSG00000049583	*Grm5*	glutamate receptor, metabotropic 5	-7.3	-3.5
8	ENSMUSG00000049939	*Lrrc4*	leucine rich repeat containing 4	-7.2	-3.5
9	ENSMUSG00000026904	*Slc4a10*	solute carrier family 4, sodium bicarbonate cotransporter-like, member 10	-7.0	-3.4
10	ENSMUSG00000039275	*Foxk2*	forkhead box K2	-7.2	-3.4
11	ENSMUSG00000037892	*Pcdh18*	protocadherin 18	-7.2	-3.3
12	ENSMUSG00000044667	*Lppr4*	lipid phosphate phosphatase-related protein type 4	-7.1	-3.3
13	ENSMUSG00000039419	*Cntnap2*	contactin associated protein-like 2	-6.6	-3.2
14	ENSMUSG00000035681	*Kcnc2*	potassium voltage gated channel, Shaw-related subfamily, member 2	-6.6	-3.2
15	ENSMUSG00000001054	*Rmnd5b*	required for meiotic nuclear division 5 homolog B (S. cerevisiae)	-6.5	-3.2
16	ENSMUSG00000078700	*D030028A08Rik*	RIKEN cDNA D030028A08 gene	-6.6	-3.2
17	ENSMUSG00000030465	*Psd3*	pleckstrin and Sec7 domain containing 3	-7.2	-3.1
18	ENSMUSG00000051451	*Crebzf*	CREB/ATF bZIP transcription factor	-6.3	-3.1
19	ENSMUSG00000045038	*Prkce*	protein kinase C, epsilon	-7.0	-3.1
20	ENSMUSG00000024269	*Tpgs2*	tubulin polyglutamylase complex subunit 2	-6.9	-3.1
21	ENSMUSG00000021196	*Pfkp*	phosphofructokinase, platelet	-6.3	-3.1
22	ENSMUSG00000031703	*Itfg1*	integrin alpha FG-GAP repeat containing 1	-5.6	-3.1
23	ENSMUSG00000040591	*1110051M20Rik*	RIKEN cDNA 1110051M20 gene	-6.6	-3.1
24	ENSMUSG00000032625	*Thsd7a*	thrombospondin, type I, domain containing 7A	-7.1	-3.0
25	ENSMUSG00000085254	*Gm15881*	predicted gene 15881	-6.4	-3.0
26	ENSMUSG00000011589	*Fsd1*	fibronectin type 3 and SPRY domain-containing protein	-6.5	-3.0
27	ENSMUSG00000025475	*Gpr123*	G protein-coupled receptor 123	-6.6	-3.0
28	ENSMUSG00000024074	*Crim1*	cysteine rich transmembrane BMP regulator 1 (chordin like)	-6.3	-3.0
29	ENSMUSG00000022151	*Ttc33*	tetratricopeptide repeat domain 33	-6.4	-3.0
30	ENSMUSG00000055435	*Maf*	avian musculoaponeurotic fibrosarcoma (v-maf) AS42 oncogene homolog	-3.8	-3.0
31	ENSMUSG00000050711	*Scg2*	secretogranin II	-6.9	-3.0
32	ENSMUSG00000005583	*Mef2c*	myocyte enhancer factor 2C	-3.7	-3.0
33	ENSMUSG00000019790	*Stxbp5*	syntaxin binding protein 5 (tomosyn)	-6.7	-3.0
34	ENSMUSG00000044288	*Cnr1*	cannabinoid receptor 1 (brain)	-3.8	-3.0
35	ENSMUSG00000026890	*Lhx6*	LIM homeobox protein 6	-6.8	-3.0
36	ENSMUSG00000063446	*Lppr1*	lipid phosphate phosphatase-related protein type 1	-6.5	-3.0
37	ENSMUSG00000029020	*Mfn2*	mitofusin 2	-6.3	-2.9
38	ENSMUSG00000053519	*Kcnip1*	Kv channel-interacting protein 1	-6.4	-2.9
39	ENSMUSG00000031176	*Dynlt3*	dynein light chain Tctex-type 3	-6.1	-2.9
40	ENSMUSG00000075595	*Zfp652*	zinc finger protein 652	-6.4	-2.9
41	ENSMUSG00000043388	*Tmem130*	transmembrane protein 130	-4.5	-2.9
42	ENSMUSG00000025228	*Actr1a*	ARP1 actin-related protein 1A, centractin alpha	-4.8	-2.9
43	ENSMUSG00000070047	*Fat1*	FAT tumor suppressor homolog 1 (Drosophila)	-6.0	-2.9
44	ENSMUSG00000048895	*Cdk5r1*	cyclin-dependent kinase 5, regulatory subunit 1 (p35)	-5.0	-2.9
45	ENSMUSG00000005672	*Kit*	kit oncogene	-6.5	-2.9
46	ENSMUSG00000061864	*Galntl6*	UDP-N-acetyl-alpha-D-galactosamine:polypeptide N-acetylgalactosaminyltransferase-like 6	-5.9	-2.9
47	ENSMUSG00000022619	*Mapk8ip2*	mitogen-activated protein kinase 8 interacting protein 2	-6.1	-2.9
48	ENSMUSG00000055409	*Nell1*	NEL-like 1	-6.0	-2.8
49	ENSMUSG00000022561	*Gpaa1*	GPI anchor attachment protein 1	-6.2	-2.8
50	ENSMUSG00000021071	*Trim9*	tripartite motif-containing 9	-6.8	-2.8
51	ENSMUSG00000066189	*Cacng3*	calcium channel, voltage-dependent, gamma subunit 3	-5.9	-2.8
52	ENSMUSG00000024109	*Nrxn1*	neurexin I	-3.8	-2.8
53	ENSMUSG00000078578	*Ube2d3*	ubiquitin-conjugating enzyme E2D 3	-4.5	-2.8
54	ENSMUSG00000031782	*Coq9*	coenzyme Q9 homolog (yeast)	-5.9	-2.8
55	ENSMUSG00000030077	*Chl1*	cell adhesion molecule with homology to L1CAM	-3.3	-2.8

56	ENSMUSG00000029174	*Tbc1d1*	TBC1 domain family, member 1	-5.9	-2.8
57	ENSMUSG00000031532	*Saraf*	store-operated calcium entry-associated regulatory factor	-4.8	-2.8
58	ENSMUSG00000048661	*Lemd3*	LEM domain containing 3	-5.9	-2.8
59	ENSMUSG00000024560	*Cxxc1*	CXXC finger 1 (PHD domain)	-6.3	-2.7
60	ENSMUSG00000040022	*Rab11fip2*	RAB11 family interacting protein 2 (class I)	-5.9	-2.7
61	ENSMUSG00000026277	*Stk25*	serine/threonine kinase 25 (yeast)	-6.4	-2.7
62	ENSMUSG00000003411	*Rab3b*	RAB3B, member RAS oncogene family	-6.4	-2.7
63	ENSMUSG00000024897	*Apba1*	amyloid beta (A4) precursor protein binding, family A, member 1	-6.0	-2.7
64	ENSMUSG00000035236	*Scai*	suppressor of cancer cell invasion	-5.1	-2.7
65	ENSMUSG00000026696	*Vamp4*	vesicle-associated membrane protein 4	-5.6	-2.7
66	ENSMUSG00000085793	*Lin52*	lin-52 homolog (C. elegans)	-6.1	-2.7
67	ENSMUSG00000029608	*Rph3a*	rabphilin 3A	-4.2	-2.7
68	ENSMUSG00000025485	*Ric8*	resistance to inhibitors of cholinesterase 8 homolog (C. elegans)	-6.3	-2.7
69	ENSMUSG00000029063	*Nadk*	NAD kinase	-6.8	-2.7
70	ENSMUSG00000046447	*Camk2n1*	calcium/calmodulin-dependent protein kinase II inhibitor 1	-5.0	-2.7
71	ENSMUSG00000031841	*Cdh13*	cadherin 13	-6.1	-2.7
72	ENSMUSG00000055373	*Fut9*	fucosyltransferase 9	-6.0	-2.7
73	ENSMUSG00000001089	*Luzp1*	leucine zipper protein 1	-5.8	-2.7
74	ENSMUSG00000020817	*Rabep1*	rabaptin, RAB GTPase binding effector protein 1	-4.9	-2.7
75	ENSMUSG00000044224	*Dnajc21*	DnaJ (Hsp40) homolog, subfamily C, member 21	-6.6	-2.7
76	ENSMUSG00000033478	*Fam160b1*	family with sequence similarity 160, member B1	-6.0	-2.7
77	ENSMUSG00000040152	*Thbs1*	thrombospondin 1	-5.9	-2.7
78	ENSMUSG00000029038	*Ssu72*	Ssu72 RNA polymerase II CTD phosphatase homolog (yeast)	-5.5	-2.7
79	ENSMUSG00000024376	*Epb4.1l4a*	erythrocyte protein band 4.1-like 4a	-5.6	-2.7
80	ENSMUSG00000018428	*Akap1*	A kinase (PRKA) anchor protein 1	-6.1	-2.7
81	ENSMUSG00000042229	*Rabif*	RAB interacting factor	-6.6	-2.6
82	ENSMUSG00000032878	*Ccdc85a*	coiled-coil domain containing 85A	-5.8	-2.6
83	ENSMUSG00000041986	*Elmod1*	ELMO/CED-12 domain containing 1	-5.0	-2.6
84	ENSMUSG00000048490	*Nrip1*	nuclear receptor interacting protein 1	-6.1	-2.6
85	ENSMUSG00000019970	*Sgk1*	serum/glucocorticoid regulated kinase 1	-5.3	-2.6
86	ENSMUSG00000084390	*Gm15425*	predicted gene 15425	-5.8	-2.6
87	ENSMUSG00000031530	*Dusp4*	dual specificity phosphatase 4	-5.6	-2.6
88	ENSMUSG00000060098	*Prmt7*	protein arginine N-methyltransferase 7	-5.8	-2.6
89	ENSMUSG00000060988	*Galnt13*	UDP-N-acetyl-alpha-D-galactosamine:polypeptide N-acetylgalactosaminyltransferase 13	-5.8	-2.6
90	ENSMUSG00000028528	*Dnajc6*	DnaJ (Hsp40) homolog, subfamily C, member 6	-5.9	-2.6
91	ENSMUSG00000030704	*Rab6a*	RAB6A, member RAS oncogene family	-4.2	-2.6
92	ENSMUSG00000060181	*Slc35e3*	solute carrier family 35, member E3	-5.6	-2.6
93	ENSMUSG00000049985	*Ankrd55*	ankyrin repeat domain 55	-5.7	-2.6
94	ENSMUSG00000070866	*Zfp804a*	zinc finger protein 804A	-5.8	-2.6
95	ENSMUSG00000026109	*Tmeff2*	transmembrane protein with EGF-like and two follistatin-like domains 2	-5.2	-2.6
96	ENSMUSG00000062906	*Hdac10*	histone deacetylase 10	-6.7	-2.6
97	ENSMUSG00000037747	*Phyhipl*	phytanoyl-CoA hydroxylase interacting protein-like	-6.1	-2.6
98	ENSMUSG00000030521	*Mphosph10*	M-phase phosphoprotein 10 (U3 small nucleolar ribonucleoprotein)	-5.3	-2.6
99	ENSMUSG00000039183	*Nubp2*	nucleotide binding protein 2	-6.0	-2.6
100	ENSMUSG00000008450	*Nutf2*	nuclear transport factor 2	-5.8	-2.6

Legend: List of genes displaying a significant down-regulation in type 1 *Htr3a*-GFP+ cortical interneurons (INs) compared to type 2 and 3 INs at P2. Genes are identified using single-cell differential display (SCDE) with a Maximum Likelihood Estimate (MLE) ≤ -2 and Z-score ≤ -2. Ranking of genes is based on Z-scores. Top 100 genes are displayed. Additional enriched genes can be found in the Supplementary data file. The gene highlighted in blue is presented in figure 1.

Supplementary Table 3: Genes up-regulated in type 2 *Htr3a*-GFP+ interneurons at P2

N°	Ensembl Identifier	Symbol	Description	Z-score	Fold Change (MLE)
1	ENSMUSG00000026904	*Slc4a10*	solute carrier family 4, sodium bicarbonate cotransporter-like, member 10	3.8	7.3
2	ENSMUSG00000040591	*1110051M20Rik*	RIKEN cDNA 1110051M20 gene	3.7	7.2
3	ENSMUSG00000035776	*Cd99l2*	CD99 antigen-like 2	3.7	7.4
4	ENSMUSG00000011589	*Fsd1*	fibronectin type 3 and SPRY domain-containing protein	3.6	6.9
5	ENSMUSG00000049939	*Lrrc4*	leucine rich repeat containing 4	3.6	7.2
6	ENSMUSG00000044288	*Cnr1*	cannabinoid receptor 1 (brain)	3.4	3.4
7	ENSMUSG00000066189	*Cacng3*	calcium channel, voltage-dependent, gamma subunit 3	3.4	6.4
8	ENSMUSG00000020053	*Igf1*	insulin-like growth factor 1	3.2	5.6
9	ENSMUSG00000039419	*Cntnap2*	contactin associated protein-like 2	3.2	6.2
10	ENSMUSG00000022295	*Atp6v1c1*	ATPase, H+ transporting, lysosomal V1 subunit C1	3.2	5.7
11	ENSMUSG00000053519	*Kcnip1*	Kv channel-interacting protein 1	3.1	7.1
12	ENSMUSG00000048490	*Nrip1*	nuclear receptor interacting protein 1	3.1	6.1
13	ENSMUSG00000030714	*Ccdc101*	coiled-coil domain containing 101	3.1	5.8
14	ENSMUSG00000050711	*Scg2*	secretogranin II	3.1	5.5
15	ENSMUSG00000039183	*Nubp2*	nucleotide binding protein 2	3.1	6.6
16	ENSMUSG00000026925	*Inpp5e*	inositol polyphosphate-5-phosphatase E	3.1	6.1
17	ENSMUSG00000070047	*Fat1*	FAT tumor suppressor homolog 1 (Drosophila)	3.0	6.0
18	ENSMUSG00000019880	*Rspo3*	R-spondin 3	3.0	5.8
19	ENSMUSG00000034825	*Nrip3*	nuclear receptor interacting protein 3	3.0	4.8
20	ENSMUSG00000027108	*Ola1*	Obg-like ATPase 1	3.0	5.9
21	ENSMUSG00000025485	*Ric8*	resistance to inhibitors of cholinesterase 8 homolog (C. elegans)	3.0	5.5
22	ENSMUSG00000024403	*Atp6v1g2*	ATPase, H+ transporting, lysosomal V1 subunit G2	3.0	5.0
23	ENSMUSG00000015247	*Nipsnap3b*	nipsnap homolog 3B (C. elegans)	3.0	6.4
24	ENSMUSG00000029174	*Tbc1d1*	TBC1 domain family, member 1	3.0	5.7
25	ENSMUSG00000003363	*Pld3*	phospholipase D family, member 3	2.9	4.4
26	ENSMUSG00000031176	*Dynlt3*	dynein light chain Tctex-type 3	2.9	6.0
27	ENSMUSG00000030086	*Chchd6*	coiled-coil-helix-coiled-coil-helix domain containing 6	2.9	5.0
28	ENSMUSG00000031242	*2610002M06Rik*	RIKEN cDNA 2610002M06 gene	2.9	5.9
29	ENSMUSG00000061864	*Galntl6*		2.9	5.5
30	ENSMUSG00000041762	*Gpr155*	G protein-coupled receptor 155	2.9	6.1
31	ENSMUSG00000024571	*Gm16286*	predicted gene 16286	2.9	5.4
32	ENSMUSG00000024897	*Apba1*	amyloid beta (A4) precursor protein binding, family A, member 1	2.9	5.9
33	ENSMUSG00000018428	*Akap1*	A kinase (PRKA) anchor protein 1	2.9	6.3
34	ENSMUSG00000010607	*Pigyl*	phosphatidylinositol glycan anchor biosynthesis, class Y-like	2.9	5.6
35	ENSMUSG00000031530	*Dusp4*	dual specificity phosphatase 4	2.8	5.6
36	ENSMUSG00000035067	*Xkr6*	X Kell blood group precursor related family member 6 homolog	2.8	5.3
37	ENSMUSG00000031467	*Agpat5*	1-acylglycerol-3-phosphate O-acyltransferase 5 (lysophosphatidic acid acyltransferase, epsilon)	2.8	5.6
38	ENSMUSG00000035681	*Kcnc2*	potassium voltage gated channel, Shaw-related subfamily, member 2	2.8	5.8
39	ENSMUSG00000043372	*Hexim2*	hexamethylene bis-acetamide inducible 2	2.8	5.2
40	ENSMUSG00000000711	*Rab5b*	RAB5B, member RAS oncogene family	2.8	5.5
41	ENSMUSG00000005583	*Mef2c*	myocyte enhancer factor 2C	2.8	3.3
42	ENSMUSG00000025041	*Nt5c2*	5'-nucleotidase, cytosolic II	2.8	5.5
43	ENSMUSG00000009628	*Tex15*	testis expressed gene 15	2.8	6.7
44	ENSMUSG00000052139	*Bre*	brain and reproductive organ-expressed protein	2.8	6.2
45	ENSMUSG00000062906	*Hdac10*	histone deacetylase 10	2.8	6.7
46	ENSMUSG00000022561	*Gpaa1*	GPI anchor attachment protein 1	2.8	6.0
47	ENSMUSG00000081670	*Gm15697*	predicted gene 15697	2.7	5.2
48	ENSMUSG00000019820	*Utrn*	utrophin	2.7	5.2
49	ENSMUSG00000042729	*Wdr74*	WD repeat domain 74	2.7	5.7
50	ENSMUSG00000008450	*Nutf2*	nuclear transport factor 2	2.7	6.1
51	ENSMUSG00000026825	*Dnm1*	dynamin 1	2.7	5.0
52	ENSMUSG00000034110	*Kctd7*	potassium channel tetramerisation domain containing 7	2.7	5.6
53	ENSMUSG00000035713	*Usp35*	ubiquitin specific peptidase 35	2.7	5.8
54	ENSMUSG00000029059	*Fam213b*	family with sequence similarity 213, member B	2.7	5.1
55	ENSMUSG00000031988	*Vps26b*	vacuolar protein sorting 26 homolog B (yeast)	2.7	5.4
56	ENSMUSG00000036699	*Zcchc12*	zinc finger, CCHC domain containing 12	2.7	3.9
57	ENSMUSG00000085254	*Gm15881*	predicted gene 15881	2.7	6.4

58	ENSMUSG00000024269	*Tpgs2*	tubulin polyglutamylase complex subunit 2	2.7	5.6
59	ENSMUSG00000063600	*Egfem1*	EGF-like and EMI domain containing 1	2.7	6.0
60	ENSMUSG00000069255	*Dusp22*	dual specificity phosphatase 22	2.6	5.5
61	ENSMUSG00000028851	*Nudc*	nuclear distribution gene C homolog (Aspergillus)	2.6	5.2
62	ENSMUSG00000033439	*Trmt13*	tRNA methyltransferase 13	2.6	5.4
63	ENSMUSG00000037499	*Nenf*	neuron derived neurotrophic factor	2.6	5.1
64	ENSMUSG00000048004	*Tmem196*	transmembrane protein 196	2.6	5.0
65	ENSMUSG00000022426	*Josd1*	Josephin domain containing 1	2.6	6.0
66	ENSMUSG00000033285	*Wdr3*	WD repeat domain 3	2.6	5.3
67	ENSMUSG00000030127	*Cops7a*	COP9 (constitutive photomorphogenic) homolog, subunit 7a (Arabidopsis thaliana)	2.6	6.1
68	ENSMUSG00000047342	*Zfp286*	zinc finger protein 286	2.6	6.6
69	ENSMUSG00000060988	*Galnt13*	UDP-N-acetyl-alpha-D-galactosamine:polypeptide N-acetylgalactosaminyltransferase 13	2.6	5.7
70	ENSMUSG00000003269	*Cyth2*	cytohesin 2	2.6	5.1
71	ENSMUSG00000026618	*Iars2*	isoleucine-tRNA synthetase 2, mitochondrial	2.6	5.7
72	ENSMUSG00000029536	*Gatc*	glutamyl-tRNA(Gln) amidotransferase, subunit C	2.6	5.2
73	ENSMUSG00000090387	*Gm17056*	predicted gene 17056	2.6	5.5
74	ENSMUSG00000022151	*Ttc33*	tetratricopeptide repeat domain 33	2.6	4.8
75	ENSMUSG00000039886	*Tmem120a*	transmembrane protein 120A	2.6	5.6
76	ENSMUSG00000042604	*Kcna4*	potassium voltage-gated channel, shaker-related subfamily, member 4	2.6	5.5
77	ENSMUSG00000053647	*Gper1*	G protein-coupled estrogen receptor 1	2.6	6.2
78	ENSMUSG00000035829	*Ppp1r26*	protein phosphatase 1, regulatory subunit 26	2.5	5.2
79	ENSMUSG00000020091	*Eif4ebp2*	eukaryotic translation initiation factor 4E binding protein 2	2.5	5.5
80	ENSMUSG00000042532	*Golga7b*	golgi autoantigen, golgin subfamily a, 7B	2.5	4.1
81	ENSMUSG00000046157	*Tmem229b*	transmembrane protein 229B	2.5	5.2
82	ENSMUSG00000021838	*Samd4*	sterile alpha motif domain containing 4	2.5	5.5
83	ENSMUSG00000003948	*Mmd*	monocyte to macrophage differentiation-associated	2.5	4.8
84	ENSMUSG00000098148	*Gm20204*		2.5	4.7
85	ENSMUSG00000037750	*Fam222b*	family with sequence similarity 222, member B	2.5	5.3
86	ENSMUSG00000032411	*Tfdp2*	transcription factor Dp 2	2.5	4.6
87	ENSMUSG00000037351	*Actr1b*	ARP1 actin-related protein 1B, centractin beta	2.5	5.8
88	ENSMUSG00000031266	*Gla*	galactosidase, alpha	2.5	4.7
89	ENSMUSG00000053716	*Dusp7*	dual specificity phosphatase 7	2.5	5.4
90	ENSMUSG00000021939	*Ctsb*	cathepsin B	2.5	4.6
91	ENSMUSG00000031198	*Fundc2*	FUN14 domain containing 2	2.5	5.8
92	ENSMUSG00000026914	*Psmd14*	proteasome (prosome, macropain) 26S subunit, non-ATPase, 14	2.5	3.1
93	ENSMUSG00000030682	*Cdipt*	CDP-diacylglycerol—inositol 3-phosphatidyltransferase (phosphatidylinositol synthase)	2.5	5.4
94	ENSMUSG00000009894	*Snap47*	synaptosomal-associated protein, 47	2.5	3.7
95	ENSMUSG00000026895	*Ndufa8*	NADH dehydrogenase (ubiquinone) 1 alpha subcomplex, 8	2.5	3.2
96	ENSMUSG00000030816	*Rnf40*	ring finger protein 40	2.5	5.6
97	ENSMUSG00000029063	*Nadk*	NAD kinase	2.5	5.1
98	ENSMUSG00000044667	*Lppr4*	lipid phosphate phosphatase-related protein type 4	2.5	3.6
99	ENSMUSG00000027472	*Pdrg1*	p53 and DNA damage regulated 1	2.5	5.4
100	ENSMUSG00000030801	*Kat8*	K(lysine) acetyltransferase 8	2.5	5.9

Legend: List of genes displaying a significant up-regulation in type 2 of *Htr3a*-GFP+ cortical interneurons (INs) compared to type 1 and 3 INs at P2. Genes are identified using single-cell differential display (SCDE) with a Maximum Likelihood Estimate (MLE) ≥ 2 and Z-score ≥ 2. Ranking of genes is based on Z-scores. Top 100 genes are displayed. Additional enriched genes can be found in the Supplementary data file.

Supplementary Table 4: Genes down-regulated in type 2 *Htr3a*-GFP+ interneurons at P2

N°	Ensembl Identifier	Symbol	Description	Z-score	Fold Change (MLE)
1	ENSMUSG00000010505	*Myt1*	myelin transcription factor 1	-6.9	-3.7
2	ENSMUSG00000029563	*Foxp2*	forkhead box P2	-6.3	-3.6
3	ENSMUSG00000020160	*Meis1*	Meis homeobox 1	-5.2	-3.5
4	ENSMUSG00000085936	*2610307P16Rik*	RIKEN cDNA 2610307P16 gene	-7.9	-3.5
5	ENSMUSG00000050558	*Prokr2*	prokineticin receptor 2	-7.7	-3.4
6	ENSMUSG00000043668	*Tox3*	TOX high mobility group box family member 3	-6.7	-3.4
7	ENSMUSG00000027210	*Meis2*	Meis homeobox 2	-4.8	-3.4
8	ENSMUSG00000023800	*Tiam2*	T cell lymphoma invasion and metastasis 2	-4.3	-3.2
9	ENSMUSG00000034614	*Pik3ip1*	phosphoinositide-3-kinase interacting protein 1	-5.8	-3.1
10	ENSMUSG00000042810	*Krba1*	KRAB-A domain containing 1	-5.2	-3.1
11	ENSMUSG00000031971	*Ccsap*	centriole, cilia and spindle associated protein	-6.9	-3.1
12	ENSMUSG00000029366	*Dck*	deoxycytidine kinase	-6.5	-3.0
13	ENSMUSG00000036737	*Oxsr1*	oxidative-stress responsive 1	-5.1	-3.0
14	ENSMUSG00000040896	*Kcnd3*	potassium voltage-gated channel, Shal-related family, member 3	-6.8	-2.9
15	ENSMUSG00000052534	*Pbx1*	pre B cell leukemia homeobox 1	-3.3	-2.9
16	ENSMUSG00000009995	*Taz*	tafazzin	-4.9	-2.8
17	ENSMUSG00000022257	*Laptm4b*	lysosomal-associated protein transmembrane 4B	-5.7	-2.8
18	ENSMUSG00000025408	*Ddit3*	DNA-damage inducible transcript 3	-5.4	-2.7
19	ENSMUSG00000025959	*Klf7*	Kruppel-like factor 7 (ubiquitous)	-3.3	-2.7
20	ENSMUSG00000049811	*Fam161a*	family with sequence similarity 161, member A	-5.6	-2.7
21	ENSMUSG00000040883	*Tmem205*	transmembrane protein 205	-6.1	-2.7
22	ENSMUSG00000000916	*Nsun5*	NOL1/NOP2/Sun domain family, member 5	-6.0	-2.7
23	ENSMUSG00000099597	*Gm28933*	predicted gene 28933	-7.1	-2.7
24	ENSMUSG00000028629	*Exo5*	exonuclease 5	-5.9	-2.6
25	ENSMUSG00000058192	*Zfp846*	zinc finger protein 846	-5.3	-2.6
26	ENSMUSG00000033705	*Stard9*	START domain containing 9	-5.0	-2.6
27	ENSMUSG00000022604	*Cep97*	centrosomal protein 97	-5.3	-2.6
28	ENSMUSG00000038718	*Pbx3*	pre B cell leukemia homeobox 3	-4.5	-2.6
29	ENSMUSG00000026766	*Mmadhc*	methylmalonic aciduria (cobalamin deficiency) cblD type, with homocystinuria	-3.4	-2.6
30	ENSMUSG00000042050	*Wdr60*	WD repeat domain 60	-6.1	-2.5
31	ENSMUSG00000027207	*Galk2*	galactokinase 2	-6.8	-2.5
32	ENSMUSG00000086308	*G630016G05Rik*	RIKEN cDNA G630016G05 gene	-5.2	-2.5
33	ENSMUSG00000053931	*Cnn3*	calponin 3, acidic	-3.6	-2.5
34	ENSMUSG00000053841	*Txlna*	taxilin alpha	-6.3	-2.5
35	ENSMUSG00000024231	*Cul2*	cullin 2	-5.0	-2.5
36	ENSMUSG00000005360	*Slc1a3*	solute carrier family 1 (glial high affinity glutamate transporter), member 3	-4.1	-2.5
37	ENSMUSG00000031512	*Tex29*	testis expressed 29	-4.8	-2.5
38	ENSMUSG00000086682	*Gm16023*	predicted gene 16023	-4.4	-2.5
39	ENSMUSG00000025702	*March8*	membrane-associated ring finger (C3HC4) 8	-5.2	-2.4
40	ENSMUSG00000021918	*Nek4*	NIMA (never in mitosis gene a)-related expressed kinase 4	-6.7	-2.4
41	ENSMUSG00000000056	*Narf*	nuclear prelamin A recognition factor	-3.7	-2.4
42	ENSMUSG00000035949	*Fbxw2*	F-box and WD-40 domain protein 2	-3.4	-2.4
43	ENSMUSG00000025733	*Rhot2*	ras homolog gene family, member T2	-5.4	-2.4
44	ENSMUSG00000022489	*Pde1b*	phosphodiesterase 1B, Ca2+-calmodulin dependent	-5.3	-2.4
45	ENSMUSG00000000184	*Ccnd2*	cyclin D2	-3.8	-2.4
46	ENSMUSG00000034612	*Chst11*	carbohydrate sulfotransferase 11	-6.6	-2.4
47	ENSMUSG00000020523	*Fam114a2*	family with sequence similarity 114, member A2	-3.1	-2.4
48	ENSMUSG00000028480	*Glipr2*	GLI pathogenesis-related 2	-5.5	-2.4
49	ENSMUSG00000027540	*Ptpn1*	protein tyrosine phosphatase, non-receptor type 1	-5.2	-2.4
50	ENSMUSG00000033326	*Kdm4a*	lysine (K)-specific demethylase 4A	-4.4	-2.4
51	ENSMUSG00000061118	*Dnajc30*	DnaJ (Hsp40) homolog, subfamily C, member 30	-4.6	-2.3
52	ENSMUSG00000104200	*RP23-172P6.1*		-4.8	-2.3
53	ENSMUSG00000004151	*Etv1*	ets variant 1	-3.1	-2.3
54	ENSMUSG00000038214	*Bend3*	BEN domain containing 3	-5.3	-2.3
55	ENSMUSG00000042063	*Zfp386*	zinc finger protein 386 (Kruppel-like)	-4.5	-2.3
56	ENSMUSG00000095139	*Pou3f2*	POU domain, class 3, transcription factor 2	-3.6	-2.3
57	ENSMUSG00000053560	*Ier2*	immediate early response 2	-3.8	-2.3
58	ENSMUSG00000102212	*RP24-146B4.2*		-3.5	-2.3
59	ENSMUSG00000040464	*Gtpbp10*	GTP-binding protein 10 (putative)	-5.7	-2.3
60	ENSMUSG00000028894	*Inpp5b*	inositol polyphosphate-5-phosphatase B	-5.2	-2.2
61	ENSMUSG00000029319	*Coq2*	coenzyme Q2 homolog, prenyltransferase (yeast)	-4.4	-2.2
62	ENSMUSG00000042439	*Zfp532*	zinc finger protein 532	-2.8	-2.2
63	ENSMUSG00000016386	*Mpped2*	metallophosphoesterase domain containing 2	-2.9	-2.2

64	ENSMUSG00000102808	*RP23-380F8.1*		-4.7	-2.2
65	ENSMUSG00000016510	*Mtif3*	mitochondrial translational initiation factor 3	-6.0	-2.2
66	ENSMUSG00000025997	*Ikzf2*	IKAROS family zinc finger 2	-3.9	-2.2
67	ENSMUSG00000080268	*Brms1*	breast cancer metastasis-suppressor 1	-4.1	-2.2
68	ENSMUSG00000005057	*Sh2b2*	SH2B adaptor protein 2	-4.3	-2.2
69	ENSMUSG00000026565	*Pou2f1*	POU domain, class 2, transcription factor 1	-3.1	-2.2
70	ENSMUSG00000040102	*Klhl42*	kelch-like 42	-6.4	-2.2
71	ENSMUSG00000022340	*Sybu*	syntabulin (syntaxin-interacting)	-6.8	-2.2
72	ENSMUSG00000056679	*Gpr173*	G-protein coupled receptor 173	-4.5	-2.2
73	ENSMUSG00000021681	*Aggf1*	angiogenic factor with G patch and FHA domains 1	-3.3	-2.2
74	ENSMUSG00000041923	*Nol4*	nucleolar protein 4	-2.5	-2.2
75	ENSMUSG00000023232	*Serinc2*	serine incorporator 2	-5.1	-2.2
76	ENSMUSG00000100561	*Gm7516*	predicted gene 7516	-4.7	-2.2
77	ENSMUSG00000027495	*Fam210b*	family with sequence similarity 210, member B	-6.2	-2.2
78	ENSMUSG00000102960	*RP24-444I15.4*		-4.9	-2.2
79	ENSMUSG00000028689	*Ccdc163*	coiled-coil domain containing 163	-5.1	-2.2
80	ENSMUSG00000021470	*Ercc6l2*	excision repair cross-complementing rodent repair deficiency, complementation group 6 like 2	-4.1	-2.1
81	ENSMUSG00000043940	*Wdfy3*	WD repeat and FYVE domain containing 3	-2.7	-2.1
82	ENSMUSG00000030314	*Atg7*	autophagy related 7	-4.4	-2.1
83	ENSMUSG00000054414	*Slc30a7*	solute carrier family 30 (zinc transporter), member 7	-4.5	-2.1
84	ENSMUSG00000097263	*Gm26804*	predicted gene, 26804	-4.2	-2.1
85	ENSMUSG00000002608	*Ccdc97*	coiled-coil domain containing 97	-4.8	-2.1
86	ENSMUSG00000032612	*Usp4*	ubiquitin specific peptidase 4 (proto-oncogene)	-2.7	-2.1
87	ENSMUSG00000038156	*Spon1*	spondin 1, (f-spondin) extracellular matrix protein	-3.6	-2.1
88	ENSMUSG00000005682	*Pan2*	PAN2 polyA specific ribonuclease subunit homolog (S. cerevisiae)	-5.6	-2.1
89	ENSMUSG00000038250	*Usp38*	ubiquitin specific peptidase 38	-3.7	-2.1
90	ENSMUSG00000026887	*Mrrf*	mitochondrial ribosome recycling factor	-5.5	-2.1
91	ENSMUSG00000028842	*Ago3*	argonaute RISC catalytic subunit 3	-3.1	-2.1
92	ENSMUSG00000060671	*Atp8b2*	ATPase, class I, type 8B, member 2	-3.7	-2.1
93	ENSMUSG00000047414	*Flrt2*	fibronectin leucine rich transmembrane protein 2	-3.6	-2.1
94	ENSMUSG00000038831	*Ralgps1*	Ral GEF with PH domain and SH3 binding motif 1	-2.6	-2.1
95	ENSMUSG00000040859	*Bsdc1*	BSD domain containing 1	-2.8	-2.1
96	ENSMUSG00000039194	*Rlbp1*	retinaldehyde binding protein 1	-3.9	-2.1
97	ENSMUSG00000033009	*Ogfod1*	2-oxoglutarate and iron-dependent oxygenase domain containing 1	-4.5	-2.1
98	ENSMUSG00000029326	*Enoph1*	enolase-phosphatase 1	-3.4	-2.1
99	ENSMUSG00000021910	*Nisch*	nischarin	-2.5	-2.1
100	ENSMUSG00000039000	*Ube3c*	ubiquitin protein ligase E3C	-3.1	-2.1

Legend: List of genes displaying a significant down-regulation in type 2 *Htr3a*-GFP+ cortical interneurons (INs) compared to type 1 and 3 (INs) at P2. Genes are identified using single-cell differential display (SCDE) with a Maximum Likelihood Estimate (MLE) ≤ -2 and Z-score ≤ -2. Ranking of genes is based on Z-scores. Top 100 genes are displayed. Additional enriched genes can be found in the Supplementary data file. The genes highlighted in red are presented in figure 1.

Supplementary Table 5: Genes up-regulated in type 3 *Htr3a*-GFP+ interneurons at P2

N°	Ensembl Identifier	Symbol	Description	Z-score	Fold Change (MLE)
1	ENSMUSG00000049866	*Arl4c*	ADP-ribosylation factor-like 4C	3.0	5.0
2	ENSMUSG00000027238	*Frmd5*	FERM domain containing 5	3.0	6.2
3	ENSMUSG00000031755	*Bbs2*	Bardet-Biedl syndrome 2 (human)	2.8	9.2
4	ENSMUSG00000018398	*Sept8*	septin 8	2.7	7.1
5	ENSMUSG00000033809	*Alg3*	asparagine-linked glycosylation 3 (alpha-1,3-mannosyltransferase)	2.6	7.6
6	ENSMUSG00000039253	*Fn3krp*	fructosamine 3 kinase related protein	2.5	5.2
7	ENSMUSG00000057069	*Ero1lb*	ERO1-like beta (S. cerevisiae)	2.4	9.0
8	ENSMUSG00000015488	*Cacfd1*	calcium channel flower domain containing 1	2.4	8.1
9	ENSMUSG00000060671	*Atp8b2*	ATPase, class I, type 8B, member 2	2.4	5.4
10	ENSMUSG00000056679	*Gpr173*	G-protein coupled receptor 173	2.4	6.3
11	ENSMUSG00000044951	*Mylk4*	myosin light chain kinase family, member 4	2.4	4.5
12	ENSMUSG00000056004	*9330182L06Rik*	RIKEN cDNA 9330182L06 gene	2.4	5.3
13	ENSMUSG00000004565	*Pnpla6*	patatin-like phospholipase domain containing 6	2.3	6.7
14	ENSMUSG00000104388	*RP24-74O18.2*	predicted gene, 37033	2.3	4.5
15	ENSMUSG00000025050	*Pcgf6*	polycomb group ring finger 6	2.3	7.2
16	ENSMUSG00000040327	*Cul9*	cullin 9	2.3	4.6
17	ENSMUSG00000060244	*Alyref2*	Aly/REF export factor 2	2.3	6.3
18	ENSMUSG00000026764	*Kif5c*	kinesin family member 5C	2.3	3.5
19	ENSMUSG00000102744	*RP24-490B17.6*	RIKEN cDNA 5830444F18 gene	2.3	6.6
20	ENSMUSG00000021238	*Aldh6a1*	aldehyde dehydrogenase family 6, subfamily A1	2.3	5.9
21	ENSMUSG00000034487	*Kdelc2*	KDEL (Lys-Asp-Glu-Leu) containing 2	2.2	4.4
22	ENSMUSG00000044847	*Lsm11*	U7 snRNP-specific Sm-like protein LSM11	2.2	5.0
23	ENSMUSG00000028955	*Vamp3*	vesicle-associated membrane protein 3	2.2	4.1
24	ENSMUSG00000074736	*Syndig1*	synapse differentiation inducing 1	2.2	9.1
25	ENSMUSG00000038495	*Otud7b*	I domain containing 7B	2.2	6.2
26	ENSMUSG00000039994	*Timeless*	timeless circadian clock 1	2.2	4.9
27	ENSMUSG00000053646	*Plxnb1*	plexin B1	2.2	5.9
28	ENSMUSG00000008859	*Rala*	v-ral simian leukemia viral oncogene homolog A (ras related)	2.2	4.0
29	ENSMUSG00000032946	*Rasgrp2*	RAS, guanyl releasing protein 2	2.1	3.7
30	ENSMUSG00000023393	*Slc17a9*	solute carrier family 17, member 9	2.1	3.6
31	ENSMUSG00000017831	*Rab5a*	RAB5A, member RAS oncogene family	2.1	5.6
32	ENSMUSG00000041911	*Dlx1*	distal-less homeobox 1	2.1	2.9
33	ENSMUSG00000089790	*Gm16588*	predicted gene 16588	2.1	5.7
34	ENSMUSG00000086896	*Cyp4x1os*	cytochrome P450, family 4, subfamily x, polypeptide 1, opposite strand	2.1	5.6
35	ENSMUSG00000021123	*Rdh12*	retinol dehydrogenase 12	2.1	4.6
36	ENSMUSG00000033400	*Agl*	amylo-1,6-glucosidase, 4-alpha-glucanotransferase	2.1	3.5
37	ENSMUSG00000061458	*Nol10*	nucleolar protein 10	2.1	5.0
38	ENSMUSG00000041263	*Rusc1*	RUN and SH3 domain containing 1	2.1	4.5
39	ENSMUSG00000096740	*Lbhd1*	LBH domain containing 1	2.1	4.4
40	ENSMUSG00000032184	*Lysmd2*	LysM, putative peptidoglycan-binding, domain containing 2	2.1	6.2
41	ENSMUSG00000049313	*Sorl1*	sortilin-related receptor, LDLR class A repeats-containing	2.1	5.4
42	ENSMUSG00000025925	*Terf1*	telomeric repeat binding factor 1	2.1	3.2
43	ENSMUSG00000028859	*Csf3r*	colony stimulating factor 3 receptor (granulocyte)	2.1	4.1
44	ENSMUSG00000026023	*Cdk15*	cyclin-dependent kinase 15	2.1	3.9
45	ENSMUSG00000028700	*Pomgnt1*	protein O-linked mannose beta 1,2-N-acetylglucosaminyltransferase	2.1	6.1
46	ENSMUSG00000050079	*Rspry1*	ring finger and SPRY domain containing 1	2.0	4.7
47	ENSMUSG00000103207	*RP23-364A24.1*	predicted gene 9874	2.0	4.0
48	ENSMUSG00000041607	*Mbp*	myelin basic protein	2.0	5.1
49	ENSMUSG00000026707	*Nsun6*	NOL1/NOP2/Sun domain family member 6	2.0	4.3
50	ENSMUSG00000064354	*mt-Co2*	mitochondrially encoded cytochrome c oxidase II	2.0	3.7
51	ENSMUSG00000104183	*RP24-408G4.3*	RIKEN cDNA 1700054O19 gene	2.0	4.3
52	ENSMUSG00000097219	*Gm26551*	predicted gene, 26551	2.0	3.5

Legend: List of genes displaying a significant up-regulation in type 3 *Htr3a*-GFP+ cortical interneurons (INs) compared to type 1 and 2 INs at P2. Genes are identified using single-cell differential display (SCDE) with a Maximum Likelihood Estimate (MLE) ≥ 2 and Z-score ≥ 2. Ranking of genes is based on Z-scores.

Supplementary Table 6: Genes down-regulated in type 3 *Htr3a*-GFP+ interneurons at P2

N°	Ensembl Identifier	Symbol	Description	Z-score	Fold Change (MLE)
1	ENSMUSG00000060036	*Rpl3*	ribosomal protein L3	-10.2	-3.5
2	ENSMUSG00000006333	*Rps9*	ribosomal protein S9	-10.2	-3.5
3	ENSMUSG00000039159	*Ube2h*	ubiquitin-conjugating enzyme E2H	-9.8	-3.3
4	ENSMUSG00000022841	*Ap2m1*	adaptor-related protein complex 2, mu 1 subunit	-7.8	-3.1
5	ENSMUSG00000021877	*Arf4*	ADP-ribosylation factor 4	-9.1	-3.0
6	ENSMUSG00000031633	*Slc25a4*	solute carrier family 25 (mitochondrial carrier, adenine nucleotide translocator), member 4	-5.8	-3.0
7	ENSMUSG00000021024	*Psma6*	proteasome (prosome, macropain) subunit, alpha type 6	-9.1	-3.0
8	ENSMUSG00000022451	*Twf1*	twinfilin, actin-binding protein, homolog 1 (Drosophila)	-9.2	-3.0
9	ENSMUSG00000032399	*Rpl4*	ribosomal protein L4	-4.8	-2.9
10	ENSMUSG00000026914	*Psmd14*	proteasome (prosome, macropain) 26S subunit, non-ATPase, 14	-8.6	-2.7
11	ENSMUSG00000004937	*Sgta*	small glutamine-rich tetratricopeptide repeat (TPR)-containing, alpha	-8.3	-2.7
12	ENSMUSG00000058355	*Abce1*	ATP-binding cassette, sub-family E (OABP), member 1	-8.4	-2.6
13	ENSMUSG00000030007	*Cct7*	chaperonin containing Tcp1, subunit 7 (eta)	-6.3	-2.6
14	ENSMUSG00000057666	*Gapdh*	glyceraldehyde-3-phosphate dehydrogenase	-8.2	-2.6
15	ENSMUSG00000034024	*Cct2*	chaperonin containing Tcp1, subunit 2 (beta)	-8.1	-2.5
16	ENSMUSG00000028495	*Rps6*	ribosomal protein S6	-8.2	-2.5
17	ENSMUSG00000025781	*Atp5c1*	ATP synthase, H+ transporting, mitochondrial F1 complex, gamma polypeptide 1	-6.7	-2.5
18	ENSMUSG00000022403	*St13*	suppression of tumorigenicity 13	-3.8	-2.5
19	ENSMUSG00000022312	*Eif3h*	eukaryotic translation initiation factor 3, subunit H	-6.6	-2.5
20	ENSMUSG00000030246	*Ldhb*	lactate dehydrogenase B	-8.1	-2.5
21	ENSMUSG00000046709	*Mapk10*	mitogen-activated protein kinase 10	-8.1	-2.5
22	ENSMUSG00000031948	*Kars*	lysyl-tRNA synthetase	-5.8	-2.4
23	ENSMUSG00000063882	*Uqcrh*	ubiquinol-cytochrome c reductase hinge protein	-7.8	-2.4
24	ENSMUSG00000023110	*Prmt5*	protein arginine N-methyltransferase 5	-7.8	-2.4
25	ENSMUSG00000022982	*Sod1*	superoxide dismutase 1, soluble	-4.4	-2.4
26	ENSMUSG00000071658	*Gng3*	guanine nucleotide binding protein (G protein), gamma 3	-5.1	-2.4
27	ENSMUSG00000024188	*Luc7l*	Luc7 homolog (S. cerevisiae)-like	-5.7	-2.4
28	ENSMUSG00000036748	*Cuedc2*	CUE domain containing 2	-7.8	-2.4
29	ENSMUSG00000022048	*Dpysl2*	dihydropyrimidinase-like 2	-2.5	-2.4
30	ENSMUSG00000030105	*Arl8b*	ADP-ribosylation factor-like 8B	-8.4	-2.4
31	ENSMUSG00000033047	*Eif3l*	eukaryotic translation initiation factor 3, subunit L	-7.8	-2.4
32	ENSMUSG00000007564	*Ppp2r1a*	protein phosphatase 2, regulatory subunit A, alpha	-4.4	-2.3
33	ENSMUSG00000015656	*Hspa8*	heat shock protein 8	-3.9	-2.3
34	ENSMUSG00000009090	*Ap1b1*	adaptor protein complex AP-1, beta 1 subunit	-7.8	-2.3
35	ENSMUSG00000024104	*Fam21*	family with sequence similarity 21	-7.6	-2.3
36	ENSMUSG00000025132	*Arhgdia*	Rho GDP dissociation inhibitor (GDI) alpha	-7.6	-2.3
37	ENSMUSG00000023944	*Hsp90ab1*	heat shock protein 90 alpha (cytosolic), class B member 1	-3.3	-2.3
38	ENSMUSG00000015776	*Med22*	mediator complex subunit 22	-7.5	-2.3
39	ENSMUSG00000039770	*Ypel5*	yippee-like 5 (Drosophila)	-5.0	-2.3
40	ENSMUSG00000016319	*Slc25a5*	solute carrier family 25 (mitochondrial carrier, adenine nucleotide translocator), member 5	-7.5	-2.3
41	ENSMUSG00000001630	*Stk38l*	serine/threonine kinase 38 like	-7.5	-2.3
42	ENSMUSG00000022553	*Maf1*	MAF1 homolog (S. cerevisiae)	-7.5	-2.2
43	ENSMUSG00000058291	*Zfp68*	zinc finger protein 68	-7.8	-2.2
44	ENSMUSG00000037935	*Smarce1*	SWI/SNF related, matrix associated, actin dependent regulator of chromatin, subfamily e, member 1	-4.6	-2.2
45	ENSMUSG00000021748	*Pdhb*	pyruvate dehydrogenase (lipoamide) beta	-7.4	-2.2
46	ENSMUSG00000016382	*Pls3*	plastin 3 (T-isoform)	-7.6	-2.2
47	ENSMUSG00000005881	*Ergic3*	ERGIC and golgi 3	-7.4	-2.2
48	ENSMUSG00000021764	*Ndufs4*	NADH dehydrogenase (ubiquinone) Fe-S protein 4	-7.4	-2.2
49	ENSMUSG00000043391	*2510009E07Rik*	RIKEN cDNA 2510009E07 gene	-7.3	-2.2
50	ENSMUSG00000029447	*Cct6a*	chaperonin containing Tcp1, subunit 6a (zeta)	-4.7	-2.2
51	ENSMUSG00000031708	*Tecr*	trans-2,3-enoyl-CoA reductase	-4.4	-2.2
52	ENSMUSG00000022956	*Atp5o*	ATP synthase, H+ transporting, mitochondrial F1 complex, O subunit	-5.8	-2.2

53	ENSMUSG00000057134	*Ado*	2-aminoethanethiol (cysteamine) dioxygenase	-4.5	-2.2
54	ENSMUSG00000025439	*Clns1a*	chloride channel, nucleotide-sensitive, 1A	-7.3	-2.2
55	ENSMUSG00000102423	*RP23-380F8.3*		-7.3	-2.2
56	ENSMUSG00000040037	*Negr1*	neuronal growth regulator 1	-7.1	-2.2
57	ENSMUSG00000043635	*Adamts3*	a disintegrin-like and metallopeptidase (reprolysin type) with thrombospondin type 1 motif, 3	-7.2	-2.1
58	ENSMUSG00000052581	*Lrrtm4*	leucine rich repeat transmembrane neuronal 4	-7.2	-2.1
59	ENSMUSG00000025156	*Gps1*	G protein pathway suppressor 1	-7.2	-2.1
60	ENSMUSG00000036707	*Cab39*	calcium binding protein 39	-7.2	-2.1
61	ENSMUSG00000042532	*Golga7b*	golgi autoantigen, golgin subfamily a, 7B	-7.3	-2.1
62	ENSMUSG00000032526	*Deb1*	differentially expressed in B16F10 1	-7.1	-2.1
63	ENSMUSG00000041997	*Tlk1*	tousled-like kinase 1	-7.2	-2.1
64	ENSMUSG00000030751	*Psma1*	proteasome (prosome, macropain) subunit, alpha type 1	-7.2	-2.1
65	ENSMUSG00000062797	*l7Rn6*	lethal, Chr 7, Rinchik 6	-7.1	-2.1
66	ENSMUSG00000022757	*Tfg*	Trk-fused gene	-7.1	-2.1
67	ENSMUSG00000022174	*Dad1*	defender against cell death 1	-7.1	-2.1
68	ENSMUSG00000027195	*Hsd17b12*	hydroxysteroid (17-beta) dehydrogenase 12	-7.2	-2.1
69	ENSMUSG00000038039	*Gcc2*	GRIP and coiled-coil domain containing 2	-7.1	-2.1
70	ENSMUSG00000061477	*Rps7*	ribosomal protein S7	-4.7	-2.1
71	ENSMUSG00000023010	*Tmbim6*	transmembrane BAX inhibitor motif containing 6	-7.1	-2.1
72	ENSMUSG00000057388	*Mrpl18*	mitochondrial ribosomal protein L18	-7.1	-2.1
73	ENSMUSG00000034825	*Nrip3*	nuclear receptor interacting protein 3	-7.0	-2.1
74	ENSMUSG00000074746	*Pdzd8*	PDZ domain containing 8	-7.1	-2.1
75	ENSMUSG00000016179	*Camk1g*	calcium/calmodulin-dependent protein kinase I gamma	-7.0	-2.1
76	ENSMUSG00000025651	*Uqcrc1*	ubiquinol-cytochrome c reductase core protein 1	-5.0	-2.1
77	ENSMUSG00000034994	*Eef2*	eukaryotic translation elongation factor 2	-3.3	-2.0
78	ENSMUSG00000030393	*Zik1*	zinc finger protein interacting with K protein 1	-7.0	-2.0
79	ENSMUSG00000014294	*Ndufa2*	NADH dehydrogenase (ubiquinone) 1 alpha subcomplex, 2	-6.9	-2.0
80	ENSMUSG00000037742	*Eef1a1*	eukaryotic translation elongation factor 1 alpha 1	-3.0	-2.0
81	ENSMUSG00000102481	*RP24-208F6.1*		-7.0	-2.0
82	ENSMUSG00000026895	*Ndufa8*	NADH dehydrogenase (ubiquinone) 1 alpha subcomplex, 8	-6.9	-2.0
83	ENSMUSG00000028184	*Lphn2*	latrophilin 2	-6.9	-2.0
84	ENSMUSG00000007659	*Bcl2l1*	BCL2-like 1	-7.1	-2.0
85	ENSMUSG00000005779	*Psmb4*	proteasome (prosome, macropain) subunit, beta type 4	-6.9	-2.0
86	ENSMUSG00000052146	*Rps10*	ribosomal protein S10	-6.9	-2.0
87	ENSMUSG00000030695	*Aldoa*	aldolase A, fructose-bisphosphate	-6.9	-2.0
88	ENSMUSG00000060739	*Nsa2*	NSA2 ribosome biogenesis homolog (S. cerevisiae)	-6.9	-2.0
89	ENSMUSG00000006728	*Cdk4*	cyclin-dependent kinase 4	-5.0	-2.0
90	ENSMUSG00000020522	*Mfap3*	microfibrillar-associated protein 3	-6.7	-2.0

Legend: List of genes displaying a significant down-regulation in type 3 of *Htr3a*-GFP+ cortical interneurons (INs) compared to type 1 and 2 INs at P2. Genes are identified using single-cell differential display (SCDE) with a Maximum Likelihood Estimate (MLE) ≤ -2 and Z-score ≤ -2. Ranking of genes is based on Z-scores.

Supplementary Table 7: Genes up-regulated in type 1 *Htr3a*-GFP+ interneurons at P5

N°	Ensembl Identifier	Symbol	Description	Z-score	Fold Change (MLE)
1	ENSMUSG00000102212	*RP24-146B4.2*	RIKEN cDNA C230085N15 gene	4.7	7.3
2	ENSMUSG00000006586	*Runx1t1*	runt-related transcription factor 1; translocated to, 1 (cyclin D-related)	4.3	4.3
3	ENSMUSG00000020029	*Nudt4*	nudix (nucleoside diphosphate linked moiety X)-type motif 4	4.3	4.8
4	ENSMUSG00000102620	*RP24-378J8.2*	predicted gene, 37675	4.2	6.4
5	ENSMUSG00000001855	*Nup214*	nucleoporin 214	4.1	5.5
6	ENSMUSG00000024044	*Epb4.1l3*	erythrocyte membrane protein band 4.1 like 3	4.0	4.4
7	ENSMUSG00000016386	*Mpped2*	metallophosphoesterase domain containing 2	3.8	4.3
8	ENSMUSG00000027210	*Meis2*	Meis homeobox 2	3.7	3.6
9	ENSMUSG00000027342	*Pcna*	proliferating cell nuclear antigen	3.7	5.6
10	ENSMUSG00000033632	*AW554918*	expressed sequence AW554918	3.6	4.8
11	ENSMUSG00000044763	*Trmt10c*	tRNA methyltransferase 10C	3.5	6.7
12	ENSMUSG00000030386	*Zfp606*	zinc finger protein 606	3.5	4.4
13	ENSMUSG00000028082	*Sh3d19*	SH3 domain protein D19	3.5	5.8
14	ENSMUSG00000007812	*Zfp655*	zinc finger protein 655	3.4	4.2
15	ENSMUSG00000041992	*Rapgef5*	Rap guanine nucleotide exchange factor (GEF) 5	3.4	4.9
16	ENSMUSG00000022241	*Tars*	threonyl-tRNA synthetase	3.4	7.5
17	ENSMUSG00000017897	*Eya2*	eyes absent 2 homolog (Drosophila)	3.4	6.1
18	ENSMUSG00000102995	*RP24-186G5.1*	RIKEN cDNA A330074H02 gene	3.4	6.5
19	ENSMUSG00000038156	*Spon1*	spondin 1, (f-spondin) extracellular matrix protein	3.4	4.4
20	ENSMUSG00000026049	*Tex30*	testis expressed 30	3.3	4.9
21	ENSMUSG00000025938	*Slco5a1*	solute carrier organic anion transporter family, member 5A1	3.3	5.9
22	ENSMUSG00000029563	*Foxp2*	forkhead box P2	3.3	4.8
23	ENSMUSG00000031950	*Gabarapl2*	gamma-aminobutyric acid (GABA) A receptor-associated protein-like 2	3.3	3.3
24	ENSMUSG00000102508	*RP23-411L6.2*	predicted gene, 37367	3.3	4.8
25	ENSMUSG00000030612	*Mrpl46*	mitochondrial ribosomal protein L46	3.2	5.6
26	ENSMUSG00000016382	*Pls3*	plastin 3 (T-isoform)	3.2	4.6
27	ENSMUSG00000023800	*Tiam2*	T cell lymphoma invasion and metastasis 2	3.2	3.4
28	ENSMUSG00000053774	*Ubxn7*	UBX domain protein 7	3.1	3.6
29	ENSMUSG00000090941	*Gm17212*	predicted gene 17212	3.1	4.6
30	ENSMUSG00000039985	*Fam60a*	family with sequence similarity 60, member A	3.1	3.2
31	ENSMUSG00000024151	*Msh2*	mutS homolog 2 (E. coli)	3.0	5.0
32	ENSMUSG00000024580	*Grpel2*	GrpE-like 2, mitochondrial	3.0	4.4
33	ENSMUSG00000067608	*Pcna-ps2*	proliferating cell nuclear antigen pseudogene 2	3.0	4.4
34	ENSMUSG00000091315	*Gm17214*	predicted gene 17214	3.0	4.8
35	ENSMUSG00000052837	*Junb*	jun B proto-oncogene	3.0	4.5
36	ENSMUSG00000036019	*Tmtc2*	transmembrane and tetratricopeptide repeat containing 2	3.0	5.1
37	ENSMUSG00000023034	*Nr4a1*	nuclear receptor subfamily 4, group A, member 1	2.9	3.7
38	ENSMUSG00000030533	*Unc45a*	unc-45 homolog A (C. elegans)	2.9	3.6
39	ENSMUSG00000065657	*Gm25337*	predicted gene, 25337	2.9	4.1
40	ENSMUSG00000052040	*Klf13*	Kruppel-like factor 13	2.9	2.5
41	ENSMUSG00000026632	*Tatdn3*	TatD Dnase domain containing 3	2.9	5.6
42	ENSMUSG00000021115	*Vrk1*	vaccinia related kinase 1	2.9	4.2
43	ENSMUSG00000032185	*Carm1*	coactivator-associated arginine methyltransferase 1	2.9	4.3
44	ENSMUSG00000025340	*Rabgef1*	RAB guanine nucleotide exchange factor (GEF) 1	2.9	3.8
45	ENSMUSG00000002732	*Fkbp7*	FK506 binding protein 7	2.9	5.2
46	ENSMUSG00000093938	*Evi2b*	ecotropic viral integration site 2b	2.9	4.1
47	ENSMUSG00000032388	*Spg21*	spastic paraplegia 21 homolog (human)	2.9	3.8
48	ENSMUSG00000071359	*Tbpl1*	TATA box binding protein-like 1	2.9	3.9
49	ENSMUSG00000024590	*Lmnb1*	lamin B1	2.9	4.8
50	ENSMUSG00000079036	*Alkbh1*	alkB, alkylation repair homolog 1 (E. coli)	2.9	3.4
51	ENSMUSG00000033166	*Dis3*	DIS3 mitotic control homolog (S. cerevisiae)	2.8	4.8
52	ENSMUSG00000022634	*Yaf2*	YY1 associated factor 2	2.8	3.9
53	ENSMUSG00000038482	*Tfdp1*	transcription factor Dp 1	2.8	4.3
54	ENSMUSG00000022120	*Rnf219*	ring finger protein 219	2.8	4.0
55	ENSMUSG00000090516	*Rps11-ps1*	ribosomal protein S11, pseudogene 1	2.8	4.9
56	ENSMUSG00000078453	*Abracl*	ABRA C-terminal like	2.8	3.5
57	ENSMUSG00000020883	*Fbxl20*	F-box and leucine-rich repeat protein 20	2.8	4.7
58	ENSMUSG00000043518	*Rai2*	retinoic acid induced 2	2.8	4.8
59	ENSMUSG00000020160	*Meis1*	Meis homeobox 1	2.8	3.3
60	ENSMUSG00000021614	*Vcan*	versican	2.8	2.7

61	ENSMUSG00000036983	*Tfb1m*	transcription factor B1, mitochondrial	2.7	4.5
62	ENSMUSG00000020752	*Recql5*	RecQ protein-like 5	2.7	5.0
63	ENSMUSG00000087590	*2410004N09Rik*	erythrocyte membrane protein band 4.1 like 4a, opposite strand	2.7	4.3
64	ENSMUSG00000038718	*Pbx3*	pre B cell leukemia homeobox 3	2.7	3.0
65	ENSMUSG00000030104	*Edem1*	ER degradation enhancer, mannosidase alpha-like 1	2.7	3.6
66	ENSMUSG00000041096	*Tspyl2*	TSPY-like 2	2.7	4.2
67	ENSMUSG00000019173	*Rab5c*	RAB5C, member RAS oncogene family	2.7	4.7
68	ENSMUSG00000087260	*Lamtor5*	late endosomal/lysosomal adaptor, MAPK and MTOR activator 5	2.7	3.3
69	ENSMUSG00000015880	*Ncapg*	non-SMC 23dducing23g I complex, subunit G	2.7	5.0
70	ENSMUSG00000102496	*RP23-461O23.2*	predicted gene, 36989	2.7	4.7
71	ENSMUSG00000001985	*Grik3*	glutamate receptor, ionotropic, 23dducin 3	2.7	3.3
72	ENSMUSG00000036478	*Btg1*	B cell translocation gene 1, anti-proliferative	2.7	2.3
73	ENSMUSG00000037993	*Dhx38*	DEAH (Asp-Glu-Ala-His) box polypeptide 38	2.7	5.4
74	ENSMUSG00000103614	*RP24-541A5.3*	predicted gene, 37949	2.7	3.4
75	ENSMUSG00000048562	*Sp8*	trans-acting transcription factor 8	2.7	2.5
76	ENSMUSG00000032562	*Gnai2*	guanine nucleotide binding protein (G protein), alpha inhibiting 2	2.6	3.2
77	ENSMUSG00000038784	*Cnot4*	CCR4-NOT transcription complex, subunit 4	2.6	3.3
78	ENSMUSG00000041923	*Nol4*	nucleolar protein 4	2.6	2.7
79	ENSMUSG00000030849	*Fgfr2*	fibroblast growth factor receptor 2	2.6	3.2
80	ENSMUSG00000032652	*Crebl2*	cAMP responsive element binding protein-like 2	2.6	3.7
81	ENSMUSG00000044164	*Rnf182*	ring finger protein 182	2.6	5.7
82	ENSMUSG00000027016	*Zfp385b*	zinc finger protein 385B	2.6	2.7
83	ENSMUSG00000042632	*Pla2g6*	phospholipase A2, group VI	2.6	4.6
84	ENSMUSG00000022009	*Nufip1*	nuclear fragile X mental retardation protein interacting protein 1	2.6	3.8
85	ENSMUSG00000087396	*4933407K13Rik*	RIKEN cDNA 4933407K13 gene	2.6	5.0
86	ENSMUSG00000028271	*Gtf2b*	general transcription factor IIB	2.6	4.4
87	ENSMUSG00000021982	*Cdadc1*	cytidine and dCMP deaminase domain containing 1	2.6	3.1
88	ENSMUSG00000027628	*Aar2*	AAR2 splicing factor homolog (S. cerevisiae)	2.6	4.2
89	ENSMUSG00000093465	*Gm20682*	predicted gene 20682	2.5	5.4
90	ENSMUSG00000087938	*Gm26049*	predicted gene, 26049	2.5	4.8
91	ENSMUSG00000046062	*Ppp1r15b*	protein phosphatase 1, regulatory (inhibitor) subunit 15b	2.5	3.7
92	ENSMUSG00000039967	*Zfp292*	zinc finger protein 292	2.5	2.5
93	ENSMUSG00000051316	*Taf7*	TAF7 RNA polymerase II, TATA box binding protein (TBP)-associated factor	2.5	2.9
94	ENSMUSG00000052423	*B4galt3*	UDP-Gal:betaGlcNAc beta 1,4-galactosyltransferase, polypeptide 3	2.5	4.2
95	ENSMUSG00000066278	*Vps37b*	vacuolar protein sorting 37B (yeast)	2.5	4.6
96	ENSMUSG00000049969	*Plekhf2*	pleckstrin homology domain containing, family F (with FYVE domain) member 2	2.5	4.4
97	ENSMUSG00000029603	*Dtx1*	deltex 1 homolog (Drosophila)	2.5	5.8
98	ENSMUSG00000026925	*Inpp5e*	inositol polyphosphate-5-phosphatase E	2.5	3.1
99	ENSMUSG00000063810	*Alms1*	Alstrom syndrome 1	2.5	4.4
100	ENSMUSG00000019782	*Rwdd1*	RWD domain containing 1	2.5	3.4

Legend: List of genes displaying a significant up-regulation in type 1 of *Htr3a*-GFP+ cortical interneurons (INs) compared to type 2 and 3 INs at P5. Genes are identified using single-cell differential display (SCDE) with a Maximum Likelihood Estimate (MLE) ≥ 2 and Z-score ≥ 2. Ranking of genes is based on Z-scores. Top 100 genes are displayed. Additional enriched genes can be found in the Supplementary data file. The genes highlighted in red are presented in figure 1.

Supplementary Table 8: Genes down-regulated in type 1 *Htr3a*-GFP+ interneurons at P5

N°	Ensembl Identifier	Symbol	Description	Z-score	Fold Change (MLE)
1	ENSMUSG00000044288	*Cnr1*	cannabinoid receptor 1 (brain)	-4.5	-5.2
2	ENSMUSG00000030077	*Chl1*	cell adhesion molecule with homology to L1CAM	-4.4	-4.7
3	ENSMUSG00000035131	*Brinp3*	bone morphogenetic protein/retinoic acid inducible neural specific 3	-7.1	-4.6
4	ENSMUSG00000022037	*Clu*	clusterin	-7.3	-4.5
5	ENSMUSG00000052726	*Kcnt2*	potassium channel, subfamily T, member 2	-7.0	-4.4
6	ENSMUSG00000046159	*Chrm3*	cholinergic receptor, muscarinic 3, cardiac	-6.5	-4.3
7	ENSMUSG00000026904	*Slc4a10*	solute carrier family 4, sodium bicarbonate cotransporter-like, member 10	-6.6	-4.3
8	ENSMUSG00000044349	*Snhg11*	small nucleolar RNA host gene 11	-4.5	-4.2
9	ENSMUSG00000029571	*Tmem106b*	transmembrane protein 106B	-6.2	-4.1
10	ENSMUSG00000028399	*Ptprd*	protein tyrosine phosphatase, receptor type, D	-4.2	-3.9
11	ENSMUSG00000043496	*Tril*	TLR4 interactor with leucine-rich repeats	-6.2	-3.9
12	ENSMUSG00000022865	*Cxadr*	coxsackie virus and adenovirus receptor	-4.8	-3.9
13	ENSMUSG00000035681	*Kcnc2*	potassium voltage gated channel, Shaw-related subfamily, member 2	-5.4	-3.8
14	ENSMUSG00000047787	*Flrt1*	fibronectin leucine rich transmembrane protein 1	-5.6	-3.8
15	ENSMUSG00000045994	*B3gat1*	beta-1,3-glucuronyltransferase 1 (glucuronosyltransferase P)	-5.2	-3.7
16	ENSMUSG00000047379	*B3gnt1*	UDP-GlcNAc:betaGal beta-1,3-N-acetylglucosaminyltransferase 1	-6.2	-3.7
17	ENSMUSG00000042401	*Crtac1*	cartilage acidic protein 1	-6.2	-3.6
18	ENSMUSG00000025026	*Add3*	24dducing 3 (gamma)	-5.7	-3.6
19	ENSMUSG00000030256	*Bhlhe41*	basic helix-loop-helix family, member e41	-5.9	-3.6
20	ENSMUSG00000027002	*Nckap1*	NCK-associated protein 1	-4.6	-3.6
21	ENSMUSG00000048834	*Vstm2a*	V-set and transmembrane domain containing 2A	-4.8	-3.5
22	ENSMUSG00000024500	*Ppp2r2b*	protein phosphatase 2, regulatory subunit B, beta	-6.0	-3.5
23	ENSMUSG00000020042	*Btbd11*	BTB (POZ) domain containing 11	-6.2	-3.5
24	ENSMUSG00000030551	*Nr2f2*	nuclear receptor subfamily 2, group F, member 2	-3.7	-3.5
25	ENSMUSG00000062098	*Btbd3*	BTB (POZ) domain containing 3	-7.0	-3.5
26	ENSMUSG00000065090	*Gm23677*	predicted gene, 23677	-5.3	-3.5
27	ENSMUSG00000030092	*Cntn6*	contactin 6	-5.6	-3.5
28	ENSMUSG00000055435	*Maf*	avian musculoaponeurotic fibrosarcoma (v-maf) AS42 oncogene homolog	-3.4	-3.5
29	ENSMUSG00000049583	*Grm5*	glutamate receptor, metabotropic 5	-4.4	-3.5
30	ENSMUSG00000078307	*AI593442*	expressed sequence AI593442	-4.3	-3.5
31	ENSMUSG00000104017	*RP23-317L18.3*		-3.5	-3.5
32	ENSMUSG00000053141	*Ptprt*	protein tyrosine phosphatase, receptor type, T	-7.0	-3.4
33	ENSMUSG00000015968	*Cacna1d*	calcium channel, voltage-dependent, L type, alpha 1D subunit	-5.6	-3.4
34	ENSMUSG00000023952	*Gtpbp2*	GTP binding protein 2	-6.4	-3.4
35	ENSMUSG00000009394	*Syn2*	synapsin II	-3.8	-3.4
36	ENSMUSG00000033419	*Snap91*	synaptosomal-associated protein 91	-3.6	-3.4
37	ENSMUSG00000058897	*Col25a1*	collagen, type XXV, alpha 1	-5.8	-3.4
38	ENSMUSG00000030259	*Rassf8*	Ras association (RalGDS/AF-6) domain family (N-terminal) member 8	-7.2	-3.4
39	ENSMUSG00000019796	*Lrp11*	low density lipoprotein receptor-related protein 11	-5.3	-3.4
40	ENSMUSG00000075318	*Scn2a1*	sodium channel, voltage-gated, type II, alpha 1	-3.7	-3.4
41	ENSMUSG00000020053	*Igf1*	insulin-like growth factor 1	-4.2	-3.3
42	ENSMUSG00000020776	*Fbf1*	Fas (TNFRSF6) binding factor 1	-5.4	-3.3
43	ENSMUSG00000056602	*Fry*	furry homolog (Drosophila)	-3.6	-3.3
44	ENSMUSG00000033382	*Trappc8*	trafficking protein particle complex 8	-5.2	-3.3
45	ENSMUSG00000029822	*Osbpl3*	oxysterol binding protein-like 3	-5.5	-3.3
46	ENSMUSG00000025889	*Snca*	synuclein, alpha	-4.5	-3.3
47	ENSMUSG00000024924	*Vldlr*	very low density lipoprotein receptor	-4.1	-3.3
48	ENSMUSG00000071424	*Grid2*	glutamate receptor, ionotropic, delta 2	-5.9	-3.3
49	ENSMUSG00000045776	*Lrtm1*	leucine-rich repeats and transmembrane domains 1	-5.0	-3.3
50	ENSMUSG00000061981	*Flot2*	flotillin 2	-5.5	-3.3
51	ENSMUSG00000039533	*Mmd2*	monocyte to macrophage differentiation-associated 2	-7.2	-3.2
52	ENSMUSG00000043456	*Zfp536*	zinc finger protein 536	-4.0	-3.2
53	ENSMUSG00000008763	*Man1a2*	mannosidase, alpha, class 1A, member 2	-3.9	-3.2
54	ENSMUSG00000069520	*Tmem19*	transmembrane protein 19	-5.1	-3.2
55	ENSMUSG00000097736	*9530059O14Rik*	RIKEN cDNA 9530059O14 gene	-5.3	-3.2

56	ENSMUSG00000055373	*Fut9*	fucosyltransferase 9	-4.1	-3.2
57	ENSMUSG00000022471	*Xrcc6*	X-ray repair complementing defective repair in Chinese hamster cells 6	-5.2	-3.2
58	ENSMUSG00000033964	*Zbtb41*	zinc finger and BTB domain containing 41 homolog	-5.0	-3.2
59	ENSMUSG00000055254	*Ntrk2*	neurotrophic tyrosine kinase, receptor, type 2	-3.4	-3.2
60	ENSMUSG00000033849	*B3galt2*	UDP-Gal:betaGlcNAc beta 1,3-galactosyltransferase, polypeptide 2	-5.9	-3.2
61	ENSMUSG00000032878	*Ccdc85a*	coiled-coil domain containing 85A	-6.4	-3.1
62	ENSMUSG00000007653	*Gabrb2*	gamma-aminobutyric acid (GABA) A receptor, subunit beta 2	-6.3	-3.1
63	ENSMUSG00000028780	*Sema3c*	sema domain, immunoglobulin domain (Ig), short basic domain, secreted, (semaphorin) 3C	-3.4	-3.1
64	ENSMUSG00000024743	*Syt7*	synaptotagmin VII	-5.0	-3.1
66	ENSMUSG00000041907	*Gpr45*	G protein-coupled receptor 45	-3.7	-3.1
67	ENSMUSG00000022935	*Grik1*	glutamate receptor, ionotropic, kainate 1	-3.9	-3.1
68	ENSMUSG00000035653	*Lrfn5*	leucine rich repeat and fibronectin type III domain containing 5	-5.1	-3.1
69	ENSMUSG00000028224	*Nbn*	nibrin	-5.2	-3.1
70	ENSMUSG00000005089	*Slc1a2*	solute carrier family 1 (glial high affinity glutamate transporter), member 2	-4.1	-3.1
71	ENSMUSG00000020810	*Cygb*	cytoglobin	-5.7	-3.1
72	ENSMUSG00000056222	*Spock1*	sparc/osteonectin, cwcv and kazal-like domains proteoglycan 1	-3.6	-3.1
73	ENSMUSG00000027099	*Mtx2*	metaxin 2	-5.6	-3.1
74	ENSMUSG00000020238	*Ncln*	nicalin homolog (zebrafish)	-4.8	-3.1
75	ENSMUSG00000022494	*Shisa9*	shisa family member 9	-4.2	-3.1
77	ENSMUSG00000034796	*Cpne7*	copine VII	-5.5	-3.0
78	ENSMUSG00000031503	*Col4a2*	collagen, type IV, alpha 2	-5.8	-3.0
79	ENSMUSG00000055026	*Gabrg3*	gamma-aminobutyric acid (GABA) A receptor, subunit gamma 3	-6.3	-3.0
80	ENSMUSG00000028360	*Slc44a5*	solute carrier family 44, member 5	-4.1	-3.0
81	ENSMUSG00000027692	*Tnik*	TRAF2 and NCK interacting kinase	-3.3	-3.0
82	ENSMUSG00000025856	*Pdgfa*	platelet derived growth factor, alpha	-4.9	-3.0
83	ENSMUSG00000045733	*Sprn*	shadow of prion protein	-5.4	-3.0
84	ENSMUSG00000031706	*Rfx1*	regulatory factor X, 1 (influences HLA class II expression)	-4.8	-3.0
85	ENSMUSG00000021820	*Camk2g*	calcium/calmodulin-dependent protein kinase II gamma	-3.6	-3.0
86	ENSMUSG00000031242	*2610002M06Rik*	RIKEN cDNA 2610002M06 gene	-5.0	-3.0
87	ENSMUSG00000031618	*Nr3c2*	nuclear receptor subfamily 3, group C, member 2	-5.3	-3.0
88	ENSMUSG00000041986	*Elmod1*	ELMO/CED-12 domain containing 1	-3.9	-3.0
89	ENSMUSG00000029608	*Rph3a*	rabphilin 3A	-3.1	-3.0
90	ENSMUSG00000024647	*Cbln2*	cerebellin 2 precursor protein	-4.1	-3.0
91	ENSMUSG00000041773	*Enc1*	ectodermal-neural cortex 1	-3.5	-3.0
92	ENSMUSG00000102657	*RP23-204I16.3*		-3.5	-3.0
93	ENSMUSG00000049281	*Scn3b*	sodium channel, voltage-gated, type III, beta	-3.8	-3.0
94	ENSMUSG00000102139	*RP24-483O2.1*		-6.2	-3.0
95	ENSMUSG00000060780	*Lrrtm1*	leucine rich repeat transmembrane neuronal 1	-4.9	-3.0
96	ENSMUSG00000020829	*Slc46a1*	solute carrier family 46, member 1	-6.2	-2.9
97	ENSMUSG00000004267	*Eno2*	enolase 2, gamma neuronal	-5.3	-2.9
98	ENSMUSG00000029156	*Sgcb*	sarcoglycan, beta (dystrophin-associated glycoprotein)	-5.4	-2.9
99	ENSMUSG00000020121	*Srgap1*	SLIT-ROBO Rho GTPase activating protein 1	-3.8	-2.9
100	ENSMUSG00000034473	*Sec22a*	SEC22 vesicle trafficking protein homolog A (S. cerevisiae)	-5.8	-2.9

Legend: List of genes displaying a significant down-regulation in type 1 *Htr3a*-GFP+ cortical interneurons (INs) compared to type 2 and 3 INs at P5. Genes are identified using single-cell differential display (SCDE) with a Maximum Likelihood Estimate (MLE) ≤ -2 and Z-score ≤ -2. Ranking of genes is based on Z-scores. Top 100 genes are displayed. The color-highlighted genes are presented in figure 1.

Supplementary Table 9: Genes up-regulated in type 2 *Htr3a*-GFP+ interneurons at P5

N°	Ensembl Identifier	Symbol	Description	Z-score	Fold Change (MLE)
1	ENSMUSG00000043496	*Tril*	TLR4 interactor with leucine-rich repeats	5.4	7.3
2	ENSMUSG00000021981	*Cab39l*	calcium binding protein 39-like	3.9	6.3
3	ENSMUSG00000026701	*Prdx6*	peroxiredoxin 6	3.8	5.2
4	ENSMUSG00000053801	*Grwd1*	glutamate-rich WD repeat containing 1	3.8	6.2
5	ENSMUSG00000037493	*Cib2*	calcium and integrin binding family member 2	3.7	5.0
6	ENSMUSG00000031723	*Txnl4b*	thioredoxin-like 4B	3.5	5.8
7	ENSMUSG00000031955	*Bcar1*	breast cancer anti-estrogen resistance 1	3.4	5.3
8	ENSMUSG00000032641	*Gpr19*	G protein-coupled receptor 19	3.4	5.0
9	ENSMUSG00000028583	*Pdpn*	podoplanin	3.3	5.5
10	ENSMUSG00000032724	*Abtb2*	ankyrin repeat and BTB (POZ) domain containing 2	3.3	6.3
11	ENSMUSG00000003868	*Ruvbl2*	RuvB-like protein 2	3.2	6.0
12	ENSMUSG00000032349	*Elovl5*	ELOVL family member 5, elongation of long chain fatty acids (yeast)	3.2	5.8
13	ENSMUSG00000024516	*Sec11c*	SEC11 homolog C, signal peptidase complex subunit	3.1	4.2
14	ENSMUSG00000028525	*Pde4b*	phosphodiesterase 4B, cAMP specific	3.1	4.9
15	ENSMUSG00000024997	*Prdx3*	peroxiredoxin 3	3.1	5.3
16	ENSMUSG00000061981	*Flot2*	flotillin 2	3.1	4.8
17	ENSMUSG00000022674	*Ube2v2*	ubiquitin-conjugating enzyme E2 variant 2	3.1	3.3
18	ENSMUSG00000033615	*Cplx1*	complexin 1	3.0	3.8
19	ENSMUSG00000062012	*Zfp13*	zinc finger protein 13	3.0	5.5
20	ENSMUSG00000033111	*3830406C13Rik*	RIKEN cDNA 3830406C13 gene	2.9	6.6
21	ENSMUSG00000035914	*Cd276*	CD276 antigen	2.9	5.1
22	ENSMUSG00000025041	*Nt5c2*	5'-nucleotidase, cytosolic II	2.9	5.3
23	ENSMUSG00000004267	*Eno2*	enolase 2, gamma neuronal	2.8	4.3
24	ENSMUSG00000022337	*Emc2*	ER membrane protein complex subunit 2	2.8	4.5
25	ENSMUSG00000017858	*Ift52*	intraflagellar transport 52	2.8	5.0
26	ENSMUSG00000056629	*Fkbp2*	FK506 binding protein 2	2.8	4.7
27	ENSMUSG00000033350	*Chst2*	carbohydrate sulfotransferase 2	2.8	5.8
28	ENSMUSG00000071379	*Hpcal1*	hippocalcin-like 1	2.8	5.9
29	ENSMUSG00000031176	*Dynlt3*	dynein light chain Tctex-type 3	2.8	5.1
30	ENSMUSG00000056204	*Pgpep1*	pyroglutamyl-peptidase I	2.8	4.8
31	ENSMUSG00000057497	*Fam136a*	family with sequence similarity 136, member A	2.8	4.0
32	ENSMUSG00000024799	*Tm7sf2*	transmembrane 7 superfamily member 2	2.7	4.8
33	ENSMUSG00000026817	*Ak1*	adenylate kinase 1	2.7	3.4
34	ENSMUSG00000056820	*Tsnax*	translin-associated factor X	2.7	3.3
35	ENSMUSG00000023572	*Ccndbp1*	cyclin D-type binding-protein 1	2.7	3.5
36	ENSMUSG00000028419	*Chmp5*	charged multivesicular body protein 5	2.7	3.0
37	ENSMUSG00000030086	*Chchd6*	coiled-coil-helix-coiled-coil-helix domain containing 6	2.7	3.8
38	ENSMUSG00000031367	*Ap1s2*	adaptor-related protein complex 1, sigma 2 subunit	2.7	2.8
39	ENSMUSG00000003363	*Pld3*	phospholipase D family, member 3	2.7	4.4
40	ENSMUSG00000031148	*Gpkow*	G patch domain and KOW motifs	2.7	4.8
41	ENSMUSG00000063600	*Egfem1*	EGF-like and EMI domain containing 1	2.7	6.0
42	ENSMUSG00000013698	*Pea15a*	phosphoprotein enriched in astrocytes 15A	2.7	3.6
43	ENSMUSG00000032252	*Glce*	glucuronyl C5-epimerase	2.6	6.6
44	ENSMUSG00000057455	*Rit2*	Ras-like without CAAX 2	2.6	4.5
45	ENSMUSG00000024253	*Dync2li1*	dynein cytoplasmic 2 light intermediate chain 1	2.6	5.5
46	ENSMUSG00000054309	*Cpsf3*	cleavage and polyadenylation specificity factor 3	2.6	3.4
47	ENSMUSG00000003161	*Sri*	sorcin	2.6	3.4
48	ENSMUSG00000008301	*Phax*	phosphorylated adaptor for RNA export	2.6	2.9
49	ENSMUSG00000068264	*Ap5s1*	adaptor-related protein 5 complex, sigma 1 subunit	2.6	5.9
50	ENSMUSG00000054408	*Spcs3*	signal peptidase complex subunit 3 homolog (S. cerevisiae)	2.6	4.9
51	ENSMUSG00000024150	*Mcfd2*	multiple coagulation factor deficiency 2	2.6	4.1
52	ENSMUSG00000103924	*RP23-184I13.4*	predicted gene, 37895	2.6	3.6
53	ENSMUSG00000095930	*Nim1k*	NIM1 serine/threonine protein kinase	2.6	5.2
54	ENSMUSG00000036790	*Slitrk2*	SLIT and NTRK-like family, member 2	2.6	3.8
55	ENSMUSG00000032121	*Tmem218*	transmembrane protein 218	2.6	5.3
56	ENSMUSG00000021607	*Mrpl36*	mitochondrial ribosomal protein L36	2.6	4.2
57	ENSMUSG00000093930	*Hmgcs1*	3-hydroxy-3-methylglutaryl-Coenzyme A synthase 1	2.6	2.7
58	ENSMUSG00000028398	*Tmem261*	transmembrane protein 261	2.6	4.3
59	ENSMUSG00000038736	*Nudcd1*	NudC domain containing 1	2.6	4.5
60	ENSMUSG00000018042	*Cyb5r3*	cytochrome b5 reductase 3	2.6	3.9
61	ENSMUSG00000009394	*Syn2*	synapsin II	2.6	2.9

62	ENSMUSG00000001785	*Pwp1*	PWP1 homolog (S. cerevisiae)	2.6	3.3
63	ENSMUSG00000032606	*Nicn1*	nicolin 1	2.5	3.0
64	ENSMUSG00000032527	*Pccb*	propionyl Coenzyme A carboxylase, beta polypeptide	2.5	4.2
65	ENSMUSG00000100691	*2010320M18Rik*	RIKEN cDNA 2010320M18 gene	2.5	4.3
66	ENSMUSG00000027447	*Cst3*	cystatin C	2.5	4.2
67	ENSMUSG00000030301	*Ccdc91*	coiled-coil domain containing 91	2.5	5.8
68	ENSMUSG00000051022	*Hs3st1*	heparan sulfate (glucosamine) 3-O-sulfotransferase 1	2.5	4.9
69	ENSMUSG00000036699	*Zcchc12*	zinc finger, CCHC domain containing 12	2.5	3.0
70	ENSMUSG00000047557	*Lxn*	latexin	2.5	4.9
71	ENSMUSG00000047044	*D030056L22Rik*	RIKEN cDNA D030056L22 gene	2.5	4.2
72	ENSMUSG00000031618	*Nr3c2*	nuclear receptor subfamily 3, group C, member 2	2.5	4.8
73	ENSMUSG00000024830	*Rps6kb2*	ribosomal protein S6 kinase, polypeptide 2	2.5	4.1
74	ENSMUSG00000034101	*Ctnnd1*	catenin (cadherin associated protein), delta 1	2.5	4.0
75	ENSMUSG00000039952	*Dag1*	dystroglycan 1	2.5	3.9
76	ENSMUSG00000032557	*Uba5*	ubiquitin-like modifier activating enzyme 5	2.5	3.5
77	ENSMUSG00000074457	*S100a16*	S100 calcium binding protein A16	2.5	5.6
78	ENSMUSG00000055760	*Gemin6*	gem (nuclear organelle) associated protein 6	2.5	4.8
79	ENSMUSG00000015944	*Gatsl2*	GATS protein-like 2	2.5	3.5
80	ENSMUSG00000060450	*Rnf14*	ring finger protein 14	2.4	3.1
81	ENSMUSG00000021929	*Kpna3*	karyopherin (importin) alpha 3	2.4	3.2
82	ENSMUSG00000002329	*Mdp1*	magnesium-dependent phosphatase 1	2.4	3.9
83	ENSMUSG00000041889	*Shisa4*	shisa family member 4	2.4	4.5
84	ENSMUSG00000084799	*Ino80dos*	INO80 complex subunit D, opposite strand	2.4	4.9
85	ENSMUSG00000035765	*Dym*	dymeclin	2.4	6.5
86	ENSMUSG00000078566	*Bnip3*	BCL2/adenovirus E1B interacting protein 3	2.4	3.9
87	ENSMUSG00000003411	*Rab3b*	RAB3B, member RAS oncogene family	2.4	4.6
88	ENSMUSG00000024427	*Spry4*	sprouty homolog 4 (Drosophila)	2.4	4.7
89	ENSMUSG00000024507	*Hsd17b4*	hydroxysteroid (17-beta) dehydrogenase 4	2.4	3.8
90	ENSMUSG00000015092	*Edf1*	endothelial differentiation-related factor 1	2.4	2.8
91	ENSMUSG00000028214	*Gem*	GTP binding protein (gene overexpressed in skeletal muscle)	2.4	6.2
92	ENSMUSG00000028191	*Bcl10*	B cell leukemia/lymphoma 10	2.4	5.6
93	ENSMUSG00000003345	*Csnk1g2*	casein kinase 1, gamma 2	2.4	3.6
94	ENSMUSG00000061374	*Fiz1*	Flt3 interacting zinc finger protein 1	2.4	5.2
95	ENSMUSG00000030772	*Dkk3*	dickkopf homolog 3 (Xenopus laevis)	2.4	4.4
96	ENSMUSG00000021013	*Ttc8*	tetratricopeptide repeat domain 8	2.4	3.1
97	ENSMUSG00000071632	*2510002D24Rik*	RIKEN cDNA 2510002D24 gene	2.4	3.6
98	ENSMUSG00000031666	*Rbl2*	retinoblastoma-like 2	2.4	3.9
99	ENSMUSG00000102344	*RP23-239I17.5*	RIKEN cDNA 9430053O09 gene	2.4	5.4
100	ENSMUSG00000029551	*Psmg3*	proteasome (prosome, macropain) assembly chaperone 3	2.4	4.1

Legend: List of genes displaying a significant over-expression in type 2 *Htr3a*-GFP+ cortical interneurons (INs) compared to type 1 and 3 INs at P5. Genes are identified using single-cell differential display (SCDE) with a Maximum Likelihood Estimate (MLE) ≥ 2 and Z-score ≥ 2. Ranking of genes is based on Z-scores. Top 100 genes are displayed. Additional enriched genes can be found in the Supplementary data file.

Supplementary Table 10: Genes down-regulated in type 2 *Htr3a*-GFP+ interneurons at P5

N°	Ensembl Identifier	Symbol	Description	Z-score	Fold Change (MLE)
1	ENSMUSG00000007812	*Zfp655*	zinc finger protein 655	-7.3	-4.5
2	ENSMUSG00000016382	*Pls3*	plastin 3 (T-isoform)	-6.8	-4.1
3	ENSMUSG00000028076	*Cd1d1*	CD1d1 antigen	-6.6	-4.0
4	ENSMUSG00000047832	*Cdca4*	cell division cycle associated 4	-6.3	-3.8
5	ENSMUSG00000102212	*RP24-146B4.2*		-6.5	-3.8
6	ENSMUSG00000030269	*Mtmr14*	myotubularin related protein 14	-6.7	-3.7
7	ENSMUSG00000006586	*Runx1t1*	runt-related transcription factor 1; translocated to, 1 (cyclin D-related)	-4.1	-3.6
8	ENSMUSG00000078676	*Casc3*	cancer susceptibility candidate 3	-6.4	-3.5
9	ENSMUSG00000020593	*Lpin1*	lipin 1	-5.9	-3.5
10	ENSMUSG00000018995	*Nars2*	asparaginyl-tRNA synthetase 2 (mitochondrial)(putative)	-5.8	-3.4
11	ENSMUSG00000033276	*Stk36*	serine/threonine kinase 36	-6.2	-3.3
12	ENSMUSG00000052406	*Rexo4*	REX4, RNA exonuclease 4 homolog (S. cerevisiae)	-6.3	-3.3
13	ENSMUSG00000001855	*Nup214*	nucleoporin 214	-5.1	-3.2
14	ENSMUSG00000030204	*Ddx47*	DEAD (Asp-Glu-Ala-Asp) box polypeptide 47	-5.2	-3.2
15	ENSMUSG00000062296	*Trank1*	tetratricopeptide repeat and ankyrin repeat containing 1	-6.0	-3.2
16	ENSMUSG00000102620	*RP24-378J8.2*		-5.6	-3.2
17	ENSMUSG00000029761	*Cald1*	caldesmon 1	-5.1	-3.1
18	ENSMUSG00000032846	*Zswim6*	zinc finger SWIM-type containing 6	-3.5	-3.1
19	ENSMUSG00000039357	*Fut11*	fucosyltransferase 11	-5.8	-3.1
20	ENSMUSG00000104094	*RP24-308L17.2*		-6.3	-3.1
21	ENSMUSG00000027016	*Zfp385b*	zinc finger protein 385B	-3.6	-3.0
22	ENSMUSG00000101778	*Gm29488*	predicted gene 29488	-5.4	-3.0
23	ENSMUSG00000048078	*Tenm4*	teneurin transmembrane protein 4	-4.5	-2.9
24	ENSMUSG00000022721	*Trmt2a*	TRM2 tRNA methyltransferase 2A	-4.8	-2.9
25	ENSMUSG00000103485	*RP23-143K19.1*		-6.1	-2.9
26	ENSMUSG00000020029	*Nudt4*	nudix (nucleoside diphosphate linked moiety X)-type motif 4	-3.9	-2.9
27	ENSMUSG00000021962	*Dcp1a*	DCP1 decapping enzyme homolog A (S. cerevisiae)	-6.3	-2.9
28	ENSMUSG00000079564	*Gm11149*	predicted gene 11149	-5.3	-2.9
29	ENSMUSG00000022241	*Tars*	threonyl-tRNA synthetase	-6.3	-2.9
30	ENSMUSG00000030747	*Dgat2*	diacylglycerol O-acyltransferase 2	-5.7	-2.9
31	ENSMUSG00000102801	*RP23-247F18.3*		-5.2	-2.9
32	ENSMUSG00000050029	*Rap2c*	RAP2C, member of RAS oncogene family	-5.0	-2.8
33	ENSMUSG00000024151	*Msh2*	mutS homolog 2 (E. coli)	-5.2	-2.8
34	ENSMUSG00000050840	*Cdh20*	cadherin 20	-5.2	-2.8
35	ENSMUSG00000048410	*Zfp407*	zinc finger protein 407	-5.3	-2.8
36	ENSMUSG00000004508	*Gab2*	growth factor receptor bound protein 2-associated protein 2	-5.2	-2.8
37	ENSMUSG00000093858	*Gm19967*	predicted gene, 19967	-5.5	-2.7
38	ENSMUSG00000020883	*Fbxl20*	F-box and leucine-rich repeat protein 20	-5.3	-2.7
39	ENSMUSG00000031792	*Usb1*	U6 snRNA biogenesis 1	-5.7	-2.7
40	ENSMUSG00000036550	*Cnot1*	CCR4-NOT transcription complex, subunit 1	-3.3	-2.7
41	ENSMUSG00000027615	*Hps3*	Hermansky-Pudlak syndrome 3 homolog (human)	-4.4	-2.7
42	ENSMUSG00000036019	*Tmtc2*	transmembrane and tetratricopeptide repeat containing 2	-5.8	-2.7
43	ENSMUSG00000028902	*Sf3a3*	splicing factor 3a, subunit 3	-4.2	-2.7
44	ENSMUSG00000006307	*Kmt2b*	lysine (K)-specific methyltransferase 2B	-4.4	-2.7
45	ENSMUSG00000029577	*Ube3b*	ubiquitin protein ligase E3B	-5.2	-2.7
46	ENSMUSG00000024044	*Epb4.1l3*	erythrocyte protein band 4.1-like 3	-3.3	-2.7
47	ENSMUSG00000028578	*Caap1*	caspase activity and apoptosis inhibitor 1	-4.9	-2.6
48	ENSMUSG00000034292	*Traf3ip1*	TRAF3 interacting protein 1	-5.3	-2.6
49	ENSMUSG00000063239	*Grm4*	glutamate receptor, metabotropic 4	-5.7	-2.6
50	ENSMUSG00000017550	*Atad5*	ATPase family, AAA domain containing 5	-4.8	-2.6
51	ENSMUSG00000020752	*Recql5*	RecQ protein-like 5	-5.1	-2.6
52	ENSMUSG00000022176	*Rem2*	rad and gem related GTP binding protein 2	-5.0	-2.6
53	ENSMUSG00000041096	*Tspyl2*	TSPY-like 2	-4.1	-2.6
54	ENSMUSG00000025533	*Asl*	argininosuccinate lyase	-4.5	-2.6
55	ENSMUSG00000017897	*Eya2*	eyes absent 2 homolog (Drosophila)	-4.8	-2.6
56	ENSMUSG00000035277	*Arx*	aristaless related homeobox	-3.0	-2.6
57	ENSMUSG00000000384	*Tbrg4*	transforming growth factor beta regulated gene 4	-4.5	-2.6
58	ENSMUSG00000090941	*Gm17212*	predicted gene 17212	-4.3	-2.6
59	ENSMUSG00000102995	*RP24-186G5.1*		-5.6	-2.5
60	ENSMUSG00000071359	*Tbpl1*	TATA box binding protein-like 1	-3.8	-2.5

61	ENSMUSG00000049672	*Zbtb14*	zinc finger and BTB domain containing 14	-5.2	-2.5
62	ENSMUSG00000004661	*Arid3b*	AT rich interactive domain 3B (BRIGHT-like)	-5.4	-2.5
63	ENSMUSG00000066440	*Zfyve26*	zinc finger, FYVE domain containing 26	-4.5	-2.5
64	ENSMUSG00000039985	*Fam60a*	family with sequence similarity 60, member A	-2.9	-2.5
65	ENSMUSG00000046961	*Gpr156*	G protein-coupled receptor 156	-5.2	-2.5
66	ENSMUSG00000024948	*Map4k2*	mitogen-activated protein kinase kinase kinase kinase 2	-4.8	-2.5
67	ENSMUSG00000045282	*Tmem86b*	transmembrane protein 86B	-5.2	-2.5
68	ENSMUSG00000091315	*Gm17214*	predicted gene 17214	-4.1	-2.5
70	ENSMUSG00000021113	*Snapc1*	small nuclear RNA activating complex, polypeptide 1	-4.9	-2.4
71	ENSMUSG00000102638	*RP23-228G18.6*		-5.3	-2.4
72	ENSMUSG00000041632	*Mrps27*	mitochondrial ribosomal protein S27	-4.4	-2.4
73	ENSMUSG00000028082	*Sh3d19*	SH3 domain protein D19	-4.8	-2.4
74	ENSMUSG00000021592	*Arsk*	arylsulfatase K	-5.1	-2.4
75	ENSMUSG00000025938	*Slco5a1*	solute carrier organic anion transporter family, member 5A1	-4.0	-2.4
76	ENSMUSG00000057229	*Atp5sl*	ATP5S-like	-5.0	-2.4
77	ENSMUSG00000029563	*Foxp2*	forkhead box P2	-3.6	-2.4
78	ENSMUSG00000020354	*Sgcd*	sarcoglycan, delta (dystrophin-associated glycoprotein)	-5.0	-2.4
79	ENSMUSG00000055491	*Pprc1*	peroxisome proliferative activated receptor, gamma, coactivator-related 1	-3.3	-2.4
80	ENSMUSG00000026632	*Tatdn3*	TatD DNase domain containing 3	-4.8	-2.4
81	ENSMUSG00000001062	*Vps9d1*	VPS9 domain containing 1	-3.6	-2.4
82	ENSMUSG00000048899	*Rimkla*	ribosomal modification protein rimK-like family member A	-4.4	-2.4
83	ENSMUSG00000102468	*RP23-133P12.2*		-4.6	-2.4
84	ENSMUSG00000047539	*Fbxo28*	F-box protein 28	-3.7	-2.3
85	ENSMUSG00000060166	*Zdhhc8*	zinc finger, DHHC domain containing 8	-4.7	-2.3
86	ENSMUSG00000051331	*Cacna1c*	calcium channel, voltage-dependent, L type, alpha 1C subunit	-2.9	-2.3
87	ENSMUSG00000038156	*Spon1*	spondin 1, (f-spondin) extracellular matrix protein	-2.9	-2.3
88	ENSMUSG00000055912	*Tmem150a*	transmembrane protein 150A	-5.2	-2.3
89	ENSMUSG00000049960	*Mrps16*	mitochondrial ribosomal protein S16	-3.9	-2.3
90	ENSMUSG00000029229	*Chic2*	cysteine-rich hydrophobic domain 2	-3.1	-2.3
91	ENSMUSG00000034813	*Grip1*	glutamate receptor interacting protein 1	-2.5	-2.3
92	ENSMUSG00000030528	*Blm*	Bloom syndrome, RecQ helicase-like	-4.4	-2.3
93	ENSMUSG00000051278	*Zgrf1*	zinc finger, GRF-type containing 1	-4.2	-2.3
94	ENSMUSG00000050075	*Gpr171*	G protein-coupled receptor 171	-4.1	-2.3
95	ENSMUSG00000024276	*Zfp397*	zinc finger protein 397	-3.4	-2.3
96	ENSMUSG00000034216	*Vps18*	vacuolar protein sorting 18 (yeast)	-4.8	-2.3
97	ENSMUSG00000063810	*Alms1*	Alstrom syndrome 1	-4.9	-2.3
98	ENSMUSG00000002732	*Fkbp7*	FK506 binding protein 7	-4.2	-2.3
99	ENSMUSG00000027342	*Pcna*	proliferating cell nuclear antigen	-3.9	-2.3
100	ENSMUSG00000040867	*Begain*	brain-enriched guanylate kinase-associated	-3.9	-2.3

Legend: List of genes displaying a significant down-regulation in type 2 *Htr3a*-GFP+ cortical interneurons (INs) compared to type 1 and 3 (INs) at P5. Genes are identified using single-cell differential display (SCDE) with a Maximum Likelihood Estimate (MLE) ≤ -2 and Z-score ≤ -2. Ranking of genes is based on Z-scores. Top 100 genes are displayed. Additional enriched genes can be found in the Supplementary data file. The gene highlighted in red is presented in figure 1.

Supplementary Table 11: Genes upregulated in type 3 *Htr3a*-GFP+ interneurons at P5

N°	Ensembl Identifier	Symbol	Description	Z-score	Fold Change (MLE)
1	ENSMUSG00000102638	*RP23-228G18.6*		4.3	8.0
2	ENSMUSG00000101609	*Kcnq1ot1*	KCNQ1 overlapping transcript 1	3.9	3.6
3	ENSMUSG00000102801	*RP23-247F18.3*	predicted gene, 37478	3.8	7.0
4	ENSMUSG00000062296	*Trank1*	tetratricopeptide repeat and ankyrin repeat containing 1	3.7	5.5
5	ENSMUSG00000022935	*Grik1*	glutamate receptor, ionotropic, kainate 1	3.7	4.9
6	ENSMUSG00000049313	*Sorl1*	sortilin-related receptor, LDLR class A repeats-containing	3.5	5.4
7	ENSMUSG00000097971	*Gm26917*	predicted gene, 26917	3.5	3.6
8	ENSMUSG00000034796	*Cpne7*	copine VII	3.4	6.5
9	ENSMUSG00000064293	*Cntn4*	contactin 4	3.4	5.4
10	ENSMUSG00000028399	*Ptprd*	protein tyrosine phosphatase, receptor type, D	3.4	3.9
11	ENSMUSG00000039057	*Myo16*	myosin XVI	3.3	6.1
12	ENSMUSG00000029088	*Kcnip4*	Kv channel interacting protein 4	3.2	5.6
13	ENSMUSG00000055254	*Ntrk2*	neurotrophic tyrosine kinase, receptor, type 2	3.2	3.6
14	ENSMUSG00000102331	*RP23-122M2.3*	predicted gene, 19938	3.1	4.5
15	ENSMUSG00000103808	*RP24-227F14.1*	predicted gene, 37060	3.1	4.9
16	ENSMUSG00000026204	*Ptprn*	protein tyrosine phosphatase, receptor type, N	3.1	4.4
17	ENSMUSG00000026163	*Sphkap*	SPHK1 interactor, AKAP domain containing	3.1	6.4
18	ENSMUSG00000049583	*Grm5*	glutamate receptor, metabotropic 5	3.0	4.1
19	ENSMUSG00000091831	*Gm4707*	predicted gene 4707	3.0	5.9
20	ENSMUSG00000036617	*Etl4*	enhancer trap locus 4	3.0	5.2
21	ENSMUSG00000033382	*Trappc8*	trafficking protein particle complex 8	3.0	4.4
22	ENSMUSG00000026153	*Fam135a*	family with sequence similarity 135, member A	3.0	4.3
23	ENSMUSG00000021196	*Pfkp*	phosphofructokinase, platelet	2.9	3.7
24	ENSMUSG00000103867	*RP23-445K23.5*	RIKEN cDNA 9630010A21 gene	2.9	4.7
25	ENSMUSG00000035131	*Brinp3*	bone morphogenetic protein/retinoic acid inducible neural specific 3	2.9	4.1
26	ENSMUSG00000071860	*2900055J20Rik*	RIKEN cDNA 2900055J20 gene	2.9	3.8
27	ENSMUSG00000044349	*Snhg11*	small nucleolar RNA host gene 11	2.8	3.3
28	ENSMUSG00000032076	*Cadm1*	cell adhesion molecule 1	2.8	3.0
29	ENSMUSG00000028780	*Sema3c*	sema domain, immunoglobulin domain (Ig), short basic domain, secreted, (semaphorin) 3C	2.8	3.5
30	ENSMUSG00000015968	*Cacna1d*	calcium channel, voltage-dependent, L type, alpha 1D subunit	2.8	4.7
31	ENSMUSG00000059146	*Ntrk3*	neurotrophic tyrosine kinase, receptor, type 3	2.8	4.3
32	ENSMUSG00000052915	*Msl1*	male-specific lethal 1 homolog (Drosophila)	2.8	3.8
33	ENSMUSG00000020866	*Cacna1g*	calcium channel, voltage-dependent, T type, alpha 1G subunit	2.8	4.2
34	ENSMUSG00000041907	*Gpr45*	G protein-coupled receptor 45	2.8	3.5
35	ENSMUSG00000078676	*Casc3*	cancer susceptibility candidate 3	2.8	4.4
36	ENSMUSG00000029822	*Osbpl3*	oxysterol binding protein-like 3	2.8	5.3
37	ENSMUSG00000024924	*Vldlr*	very low density lipoprotein receptor	2.8	3.8
38	ENSMUSG00000030249	*Abcc9*	ATP-binding cassette, sub-family C (CFTR/MRP), member 9	2.8	3.0
39	ENSMUSG00000074064	*Mlycd*	malonyl-CoA decarboxylase	2.7	6.1
40	ENSMUSG00000021991	*Cacna2d3*	calcium channel, voltage-dependent, alpha2/delta subunit 3	2.7	4.1
41	ENSMUSG00000031438	*Rnf128*	ring finger protein 128	2.7	7.4
42	ENSMUSG00000024172	*St6gal2*	beta galactoside alpha 2,6 sialyltransferase 2	2.7	4.1
43	ENSMUSG00000025326	*Ube3a*	ubiquitin protein ligase E3A	2.7	3.0
44	ENSMUSG00000072501	*Phf20l1*	PHD finger protein 20-like 1	2.7	3.5
45	ENSMUSG00000026833	*Olfm1*	olfactomedin 1	2.7	2.9
46	ENSMUSG00000055409	*Nell1*	NEL-like 1	2.7	5.4
47	ENSMUSG00000045083	*Lingo2*	leucine rich repeat and Ig domain containing 2	2.7	4.8
48	ENSMUSG00000026656	*Fcgr2b*	Fc receptor, IgG, low affinity IIb	2.7	4.8
49	ENSMUSG00000084859	*1700080N15Rik*	RIKEN cDNA 1700080N15 gene	2.7	5.3
50	ENSMUSG00000001062	*Vps9d1*	VPS9 domain containing 1	2.7	4.7
51	ENSMUSG00000052551	*Adarb2*	adenosine deaminase, RNA-specific, B2	2.6	2.8
52	ENSMUSG00000047242	*Taf9b*	TAF9B RNA polymerase II, TATA box binding protein (TBP)-associated factor	2.6	7.1
53	ENSMUSG00000032878	*Ccdc85a*	coiled-coil domain containing 85A	2.6	6.2
54	ENSMUSG00000096780	*Tmem181b-ps*	transmembrane protein 181B, pseudogene	2.6	2.5
55	ENSMUSG00000096054	*Syne1*	spectrin repeat containing, nuclear envelope 1	2.6	3.4
56	ENSMUSG00000045776	*Lrtm1*	leucine-rich repeats and transmembrane domains 1	2.6	3.8
57	ENSMUSG00000020042	*Btbd11*	BTB (POZ) domain containing 11	2.6	5.5
58	ENSMUSG00000039683	*Sdk1*	sidekick homolog 1 (chicken)	2.6	5.6
59	ENSMUSG00000016150	*Tenm1*	teneurin transmembrane protein 1	2.6	3.5
60	ENSMUSG00000032267	*Usp28*	ubiquitin specific peptidase 28	2.6	2.9

61	ENSMUSG00000056073	*Grik2*	glutamate receptor, ionotropic, kainate 2 (beta 2)	2.6	3.9
62	ENSMUSG00000030551	*Nr2f2*	nuclear receptor subfamily 2, group F, member 2	2.6	2.7
63	ENSMUSG00000102481	*RP24-208F6.1*	predicted gene, 37925	2.5	4.7
64	ENSMUSG00000035653	*Lrfn5*	leucine rich repeat and fibronectin type III domain containing 5	2.5	5.2
65	ENSMUSG00000026904	*Slc4a10*	solute carrier family 4, sodium bicarbonate cotransporter-like, member 10	2.5	4.1
66	ENSMUSG00000027677	*Ttc14*	tetratricopeptide repeat domain 14	2.5	3.4
67	ENSMUSG00000030092	*Cntn6*	contactin 6	2.5	4.4
68	ENSMUSG00000029156	*Sgcb*	sarcoglycan, beta (dystrophin-associated glycoprotein)	2.5	4.6
69	ENSMUSG00000026349	*Ccnt2*	cyclin T2	2.5	3.2
70	ENSMUSG00000027238	*Frmd5*	FERM domain containing 5	2.5	5.0
71	ENSMUSG00000038048	*Cntnap5c*	contactin associated protein-like 5C	2.5	6.5
72	ENSMUSG00000060090	*Rp2h*	retinitis pigmentosa 2 homolog (human)	2.5	3.8
73	ENSMUSG00000031441	*Atp11a*	ATPase, class VI, type 11A	2.5	3.8
74	ENSMUSG00000033061	*Resp18*	regulated endocrine-specific protein 18	2.5	5.1
75	ENSMUSG00000002763	*Pex6*	peroxisomal biogenesis factor 6	2.4	6.5
76	ENSMUSG00000021752	*Kctd6*	potassium channel tetramerisation domain containing 6	2.4	4.0
77	ENSMUSG00000101966	*Gm5248*	predicted gene 5248	2.4	3.4
78	ENSMUSG00000027716	*Trpc3*	transient receptor potential cation channel, subfamily C, member 3	2.4	5.2
79	ENSMUSG00000030350	*Prmt8*	protein arginine N-methyltransferase 8	2.4	4.7
80	ENSMUSG00000020431	*Adcy1*	adenylate cyclase 1	2.4	3.0
81	ENSMUSG00000064329	*Scn1a*	sodium channel, voltage-gated, type I, alpha	2.4	3.9
82	ENSMUSG00000102386	*RP23-184I13.7*	RIKEN cDNA 2900022M07 gene	2.4	4.4
83	ENSMUSG00000039037	*St6galnac5*	ST6 (alpha-N-acetyl-neuraminyl-2,3-beta-galactosyl-1,3)-N-acetylgalactosaminide alpha-2,6-sialyltransferase 5	2.4	4.1
84	ENSMUSG00000064341	*mt-Nd1*	mitochondrially encoded NADH dehydrogenase 1	2.4	2.0
85	ENSMUSG00000026131	*Dst*	dystonin	2.4	3.2
86	ENSMUSG00000031833	*Mast3*	microtubule associated serine/threonine kinase 3	2.4	4.8
87	ENSMUSG00000086503	*Xist*	inactive X specific transcripts	2.4	2.7
88	ENSMUSG00000042590	*Ipo11*	importin 11	2.4	4.0
89	ENSMUSG00000050075	*Gpr171*	G protein-coupled receptor 171	2.4	5.7
90	ENSMUSG00000027692	*Tnik*	TRAF2 and NCK interacting kinase	2.4	3.0
91	ENSMUSG00000056222	*Spock1*	sparc/osteonectin, cwcv and kazal-like domains proteoglycan 1	2.4	2.8
92	ENSMUSG00000098202	*B830012L14Rik*	RIKEN cDNA B830012L14 gene	2.4	4.6
93	ENSMUSG00000101776	*Gm28268*	predicted gene 28268	2.4	2.5
94	ENSMUSG00000038576	*Susd4*	sushi domain containing 4	2.4	4.1
95	ENSMUSG00000042156	*Dzip1*	DAZ interacting protein 1	2.4	3.1
96	ENSMUSG00000047789	*Slc38a9*	solute carrier family 38, member 9	2.3	4.6
97	ENSMUSG00000029095	*Ablim2*	actin-binding LIM protein 2	2.3	6.7
98	ENSMUSG00000030077	*Chl1*	cell adhesion molecule with homology to L1CAM	2.3	2.3
99	ENSMUSG00000104377	*RP23-431L24.2*	predicted gene, 37515	2.3	2.2
100	ENSMUSG00000015377	*Dennd6b*	DENN/MADD domain containing 6B	2.3	3.9

Legend: List of genes displaying a significant over-expression in type 3 *Htr3a*-GFP+ cortical interneurons (INs) compared to type 1 and 2 INs at P5. Genes are identified using single-cell differential display (SCDE) with a Maximum Likelihood Estimate (MLE) ≥ 2 and Z-score ≥ 2. Ranking of genes is based on Z-scores. Top 100 genes are displayed. Additional enriched genes can be found in the Supplementary data file. The gene highlighted in green is presented in figure 1.

Supplementary Table 12: Genes down-regulated in type 3 *Htr3a*-GFP+ interneurons at P5

N°	Ensembl Identifier	Symbol	Description	Z-score	Fold Change (MLE)
1	ENSMUSG00000004151	*Etv1*	ets variant 1	-8.9	-4.7
2	ENSMUSG00000031948	*Kars*	lysyl-tRNA synthetase	-8.8	-4.6
3	ENSMUSG00000052040	*Klf13*	Kruppel-like factor 13	-7.1	-4.5
4	ENSMUSG00000015932	*Dstn*	destrin	-7.2	-4.4
5	ENSMUSG00000004937	*Sgta*	small glutamine-rich tetratricopeptide repeat (TPR)-containing, alpha	-8.0	-4.4
6	ENSMUSG00000053536	*Cstf2t*	cleavage stimulation factor, 3' pre-RNA subunit 2, tau	-7.8	-4.3
7	ENSMUSG00000024190	*Dusp1*	dual specificity phosphatase 1	-7.9	-4.3
8	ENSMUSG00000005846	*Rsl1d1*	ribosomal L1 domain containing 1	-8.4	-4.3
9	ENSMUSG00000016386	*Mpped2*	metallophosphoesterase domain containing 2	-7.6	-4.2
10	ENSMUSG00000056515	*Rab31*	RAB31, member RAS oncogene family	-7.9	-4.1
11	ENSMUSG00000026021	*Sumo1*	SMT3 suppressor of mif two 3 homolog 1 (yeast)	-8.3	-4.1
12	ENSMUSG00000038156	*Spon1*	spondin 1, (f-spondin) extracellular matrix protein	-8.3	-4.1
13	ENSMUSG00000062661	*Ncs1*	neuronal calcium sensor 1	-5.9	-4.1
14	ENSMUSG00000041235	*Chd7*	chromodomain helicase DNA binding protein 7	-7.9	-4.1
15	ENSMUSG00000020949	*Fkbp3*	FK506 binding protein 3	-7.4	-4.1
16	ENSMUSG00000014769	*Psmb1*	proteasome (prosome, macropain) subunit, beta type 1	-7.6	-4.1
17	ENSMUSG00000027195	*Hsd17b12*	hydroxysteroid (17-beta) dehydrogenase 12	-7.7	-3.9
18	ENSMUSG00000051316	*Taf7*	TAF7 RNA polymerase II, TATA box binding protein (TBP)-associated factor	-7.1	-3.9
19	ENSMUSG00000027210	*Meis2*	Meis homeobox 2	-5.9	-3.9
20	ENSMUSG00000030246	*Ldhb*	lactate dehydrogenase B	-7.3	-3.9
21	ENSMUSG00000052684	*Jun*	jun proto-oncogene	-7.7	-3.9
22	ENSMUSG00000023800	*Tiam2*	T cell lymphoma invasion and metastasis 2	-6.1	-3.8
23	ENSMUSG00000024966	*Stip1*	stress-induced phosphoprotein 1	-7.0	-3.8
24	ENSMUSG00000050910	*Cdr2l*	cerebellar degeneration-related protein 2-like	-7.7	-3.8
25	ENSMUSG00000002379	*Ndufa11*	NADH dehydrogenase (ubiquinone) 1 alpha subcomplex 11	-6.8	-3.8
26	ENSMUSG00000027998	*Plrg1*	pleiotropic regulator 1, PRL1 homolog (Arabidopsis)	-7.2	-3.8
27	ENSMUSG00000001783	*Rtcb*	RNA 2',3'-cyclic phosphate and 5'-OH ligase	-7.8	-3.7
28	ENSMUSG00000058625	*Gm17383*	predicted gene, 17383	-6.8	-3.7
29	ENSMUSG00000015671	*Psma2*	proteasome (prosome, macropain) subunit, alpha type 2	-7.0	-3.7
30	ENSMUSG00000028282	*Casp8ap2*	caspase 8 associated protein 2	-6.9	-3.7
31	ENSMUSG00000025484	*Bet1l*	blocked early in transport 1 homolog (S. cerevisiae)-like	-6.8	-3.6
32	ENSMUSG00000024026	*Glo1*	glyoxalase 1	-7.0	-3.6
33	ENSMUSG00000005656	*Snx6*	sorting nexin 6	-6.7	-3.6
34	ENSMUSG00000031540	*Kat6a*	K(lysine) acetyltransferase 6A	-7.2	-3.6
35	ENSMUSG00000024844	*Banf1*	barrier to autointegration factor 1	-7.1	-3.6
36	ENSMUSG00000024181	*Mrpl28*	mitochondrial ribosomal protein L28	-7.0	-3.6
37	ENSMUSG00000024847	*Aip*	aryl-hydrocarbon receptor-interacting protein	-6.5	-3.5
38	ENSMUSG00000038418	*Egr1*	early growth response 1	-4.7	-3.5
39	ENSMUSG00000026313	*Hdac4*	histone deacetylase 4	-5.6	-3.5
40	ENSMUSG00000054408	*Spcs3*	signal peptidase complex subunit 3 homolog (S. cerevisiae)	-6.8	-3.5
41	ENSMUSG00000025066	*Sfr1*	SWI5 dependent recombination repair 1	-6.7	-3.5
42	ENSMUSG00000030057	*Cnbp*	cellular nucleic acid binding protein	-5.1	-3.5
43	ENSMUSG00000021690	*Jmy*	junction-mediating and regulatory protein	-6.6	-3.5
44	ENSMUSG00000032745	*Gpbp1*	GC-rich promoter binding protein 1	-6.4	-3.5
45	ENSMUSG00000035726	*Supt16*	suppressor of Ty 16	-5.2	-3.5
46	ENSMUSG00000016495	*Plgrkt*	plasminogen receptor, C-terminal lysine transmembrane protein	-6.3	-3.5
47	ENSMUSG00000071644	*Eef1g*	eukaryotic translation elongation factor 1 gamma	-5.5	-3.5
48	ENSMUSG00000030226	*Lmo3*	LIM domain only 3	-5.8	-3.5
49	ENSMUSG00000030245	*Golt1b*	golgi transport 1 homolog B (S. cerevisiae)	-5.1	-3.5
50	ENSMUSG00000029670	*Ing3*	inhibitor of growth family, member 3	-6.6	-3.4
51	ENSMUSG00000016252	*Atp5e*	ATP synthase, H+ transporting, mitochondrial F1 complex, epsilon subunit	-6.7	-3.4
52	ENSMUSG0000028419	*Chmp5*	charged multivesicular body protein 5	-7.0	-3.4
53	ENSMUSG00000032375	*Aph1b*	anterior pharynx defective 1b homolog (C. elegans)	-6.4	-3.4
54	ENSMUSG00000028541	*B4galt2*	UDP-Gal:betaGlcNAc beta 1,4-galactosyltransferase, polypeptide 2	-6.3	-3.4

55	ENSMUSG00000004849	*Ap1s1*	adaptor protein complex AP-1, sigma 1	-6.4	-3.4
56	ENSMUSG00000084159	*Gm12696*	predicted gene 12696	-6.2	-3.4
57	ENSMUSG00000023034	*Nr4a1*	nuclear receptor subfamily 4, group A, member 1	-6.9	-3.4
58	ENSMUSG00000027170	*Eif3m*	eukaryotic translation initiation factor 3, subunit M	-6.8	-3.4
59	ENSMUSG00000008301	*Phax*	phosphorylated adaptor for RNA export	-6.7	-3.3
60	ENSMUSG00000020423	*Btg2*	B cell translocation gene 2, anti-proliferative	-7.3	-3.3
61	ENSMUSG00000090862	*Rps13*	ribosomal protein S13	-5.8	-3.3
62	ENSMUSG00000060992	*Copz1*	coatomer protein complex, subunit zeta 1	-6.5	-3.3
63	ENSMUSG00000031785	*Gpr56*	G protein-coupled receptor 56	-6.6	-3.3
64	ENSMUSG00000028756	*Pink1*	PTEN induced putative kinase 1	-6.3	-3.3
65	ENSMUSG00000026826	*Nr4a2*	nuclear receptor subfamily 4, group A, member 2	-6.5	-3.3
66	ENSMUSG00000027342	*Pcna*	proliferating cell nuclear antigen	-6.4	-3.3
67	ENSMUSG00000021929	*Kpna3*	karyopherin (importin) alpha 3	-6.7	-3.3
68	ENSMUSG00000029247	*Paics*	phosphoribosylaminoimidazole carboxylase, phosphoribosylaminoribosylaminoimidazole, succinocarboxamide synthetase	-7.0	-3.3
69	ENSMUSG00000024580	*Grpel2*	GrpE-like 2, mitochondrial	-6.5	-3.3
70	ENSMUSG00000063524	*Eno1*	enolase 1, alpha non-neuron	-6.0	-3.3
71	ENSMUSG00000016427	*Ndufa1*	NADH dehydrogenase (ubiquinone) 1 alpha subcomplex, 1	-6.6	-3.3
72	ENSMUSG00000045071	*E130308A19Rik*	RIKEN cDNA E130308A19 gene	-6.4	-3.3
73	ENSMUSG00000017188	*Coa3*	cytochrome C oxidase assembly factor 3	-6.9	-3.2
74	ENSMUSG00000003299	*Mrpl4*	mitochondrial ribosomal protein L4	-6.0	-3.2
75	ENSMUSG00000015804	*Med28*	mediator complex subunit 28	-6.9	-3.2
76	ENSMUSG00000056962	*Jmjd6*	jumonji domain containing 6	-6.5	-3.2
77	ENSMUSG00000029388	*Eif2b1*	eukaryotic translation initiation factor 2B, subunit 1 (alpha)	-6.6	-3.2
78	ENSMUSG00000038069	*Cdkn2aip*	CDKN2A interacting protein	-5.9	-3.2
79	ENSMUSG00000102212	*RP24-146B4.2*		-6.4	-3.2
80	ENSMUSG00000022477	*Aco2*	aconitase 2, mitochondrial	-6.5	-3.2
81	ENSMUSG00000040822	*1700123O20Rik*	RIKEN cDNA 1700123O20 gene	-5.9	-3.2
82	ENSMUSG00000022634	*Yaf2*	YY1 associated factor 2	-6.4	-3.2
83	ENSMUSG00000031985	*Gnpat*	glyceronephosphate O-acyltransferase	-6.1	-3.2
84	ENSMUSG00000021643	*Serf1*	small EDRK-rich factor 1	-6.6	-3.2
85	ENSMUSG00000030315	*Vgll4*	vestigial like 4 (Drosophila)	-5.8	-3.2
86	ENSMUSG00000052837	*Junb*	jun B proto-oncogene	-5.8	-3.2
87	ENSMUSG00000033632	*AW554918*	expressed sequence AW554918	-5.9	-3.2
88	ENSMUSG00000053560	*Ier2*	immediate early response 2	-5.9	-3.2
89	ENSMUSG00000034210	*Efcab14*	EF-hand calcium binding domain 14	-6.4	-3.2
90	ENSMUSG00000034189	*Hsdl1*	hydroxysteroid dehydrogenase like 1	-4.5	-3.1
91	ENSMUSG00000028654	*Mycl*	v-myc myelocytomatosis viral oncogene homolog, lung carcinoma derived (avian)	-6.4	-3.1
92	ENSMUSG00000040852	*Plekhh2*	pleckstrin homology domain containing, family H (with MyTH4 domain) member 2	-6.6	-3.1
93	ENSMUSG00000025340	*Rabgef1*	RAB guanine nucleotide exchange factor (GEF) 1	-6.3	-3.1
94	ENSMUSG00000028187	*Rpf1*	ribosome production factor 1 homolog (S. cerevisiae)	-5.8	-3.1
95	ENSMUSG00000038712	*Fam63a*	family with sequence similarity 63, member A	-6.8	-3.1
96	ENSMUSG00000004667	*Polr2e*	polymerase (RNA) II (DNA directed) polypeptide E	-6.2	-3.1
97	ENSMUSG00000055720	*Ubl7*	ubiquitin-like 7 (bone marrow stromal cell-derived)	-5.7	-3.1
98	ENSMUSG00000021891	*Mettl6*	methyltransferase like 6	-5.9	-3.1
99	ENSMUSG00000036131	*Frmd7*	FERM domain containing 7	-5.9	-3.1
100	ENSMUSG00000031628	*Casp3*	caspase 3	-6.3	-3.1

Legend: List of genes displaying a significant down-regulation in type 3 *Htr3a*-GFP+ cortical interneurons (INs) compared to type 1 and 2 INs at P5. Genes are identified using single-cell differential display (SCDE) with a Maximum Likelihood Estimate (MLE) ≤ -2 and Z-score ≤ -2. Ranking of genes is based on Z-scores. Top 100 genes are displayed. Additional enriched genes can be found in the Supplementary data file. The genes highlighted in red are presented in figure 1.

Supplementary Table 13: Genes up-regulated in types of *Htr3a*-GFP+ interneurons at P2 and P5

Type 1							
N°	Ensembl Identifier	Symbol	Description	Z-score		Fold change	
				P2	P5	P2	P5
1	ENSMUSG00000023800	*Tiam2*	T cell lymphoma invasion and metastasis 2	4.1	3.2	5.2	3.4
2	ENSMUSG00000027210	*Meis2*	Meis homeobox 2	3.1	3.7	4.2	3.6
3	ENSMUSG00000029563	*Foxp2*	forkhead box P2	3.3	3.3	6.2	4.8
4	ENSMUSG00000038718	*Pbx3*	pre B cell leukemia homeobox 3	3.3	2.7	5.5	3.0
5	ENSMUSG00000038156	*Spon1*	spondin 1, (f-spondin) extracellular matrix protein	2.6	3.4	4.5	4.4
6	ENSMUSG00000052534	*Pbx1*	pre B cell leukemia homeobox 1	3.0	2.5	3.5	2.3
7	ENSMUSG00000020160	*Meis1*	Meis homeobox 1	2.7	2.8	4.3	3.3
8	ENSMUSG00000085936	*2610307P16Rik*	RIKEN cDNA 2610307P16 gene	3.3	2.1	7.6	2.4
9	ENSMUSG00000028629	*Exo5*	exonuclease 5	3.1	2.3	6.5	3.7
10	ENSMUSG00000004151	*Etv1*	ets variant 1	2.7	2.4	4.0	2.6
11	ENSMUSG00000052684	*Jun*	jun proto-oncogene	2.7	2.3	3.0	2.5
12	ENSMUSG00000043668	*Tox3*	TOX high mobility group box family member 3	2.6	2.2	5.2	3.0
13	ENSMUSG00000086308	*G630016G05Rik*	RIKEN cDNA G630016G05 gene	2.7	2.0	5.5	3.3
14	ENSMUSG00000041923	*Nol4*	nucleolar protein 4	2.0	2.6	2.3	2.7
15	ENSMUSG00000004085	*Zak*	sterile alpha motif and leucine zipper containing kinase AZK	2.0	2.2	4.0	2.8
**Type 2**							
N°	Ensembl Identifier	Symbol	Description	Z-score		Fold change	
				P2	P5	P2	P5
1	ENSMUSG00000031176	*Dynlt3*	dynein light chain Tctex-type 3	2.9	2.8	6.0	5.1
2	ENSMUSG00000031955	*Bcar1*	breast cancer anti-estrogen resistance 1	2.3	3.4	5.4	5.3
3	ENSMUSG00000003363	*Pld3*	phospholipase D family, member 3	2.9	2.7	4.4	4.4
4	ENSMUSG00000030086	*Chchd6*	coiled-coil-helix-coiled-coil-helix domain containing 6	2.9	2.7	5.0	3.8
5	ENSMUSG00000025041	*Nt5c2*	5'-nucleotidase, cytosolic II	2.8	2.9	5.5	5.3
6	ENSMUSG00000061981	*Flot2*	flotillin 2	2.4	3.1	5.6	4.8
7	ENSMUSG00000063600	*Egfem1*	EGF-like and EMI domain containing 1	2.7	2.7	6.0	6.0
8	ENSMUSG00000036699	*Zcchc12*	zinc finger, CCHC domain containing 12	2.7	2.5	3.9	3.0
9	ENSMUSG00000052139	*Bre*	brain and reproductive organ-expressed protein	2.8	2.1	6.2	3.9
10	ENSMUSG00000026895	*Ndufa8*	NADH dehydrogenase (ubiquinone) 1 alpha subcomplex, 8	2.5	2.3	3.2	2.6
11	ENSMUSG00000031367	*Ap1s2*	adaptor-related protein complex 1, sigma 2 subunit	2.1	2.7	2.5	2.8
12	ENSMUSG00000030256	*Bhlhe41*	basic helix-loop-helix family, member e41	2.2	2.3	3.5	3.3
13	ENSMUSG00000038615	*Nfe2l1*	nuclear factor, erythroid derived 2,-like 1	2.1	2.2	3.6	2.8
14	ENSMUSG00000032353	*Tmed3*	transmembrane emp24 domain containing 3	2.1	2.0	5.3	4.8
**Type 3**							
N°	Ensembl Identifier	Symbol	Description	Z-score		Fold change	
				P2	P5	P2	P5
1	ENSMUSG00000049313	*Sorl1*	sortilin-related receptor, LDLR class A repeats-containing	2.1	3.5	5.4	3.4
2	ENSMUSG00000027238	*Frmd5*	FERM domain containing 5	3.0	2.5	6.2	2.6
3	ENSMUSG00000049866	*Arl4c*	ADP-ribosylation factor-like 4C	3.0	2.3	5.0	3.6
4	ENSMUSG00000026764	*Kif5c*	kinesin family member 5C	2.3	2.3	3.5	2.8
5	ENSMUSG00000044951	*Mylk4*	myosin light chain kinase family, member 4	2.4	2.1	4.5	3.3
6	ENSMUSG00000104183	*RP24-408G4.3*	RIKEN cDNA 1700054O19 gene	2.0	2.2	4.3	3.7

Legend: List of genes displaying a significant over-expression in types of *Htr3a*-GFP+ cortical interneurons at P2 and P5. Genes are identified using single-cell differential display (SCDE) with a Maximum Likelihood Estimate (MLE) ≥ 2 and Z-score ≥ 2. Ranking of genes is based on Z-scores. The genes highlighted in red are presented in figure 1.

Supplementary Table 14: Cells counts and number of brains used for quantifications

Age	Region analyzed	Brains	Type of cell analyzed	Cells	Colocalisation quantification	Shown in
E14	Caudal ganglionic eminence (CGE)	n=3	*Htr3a*-GFP$^+$	n=2541	COUPTFII	Figure 5d
				n=4024	MEIS2/PROX1	Figure 4a
	Pallial Subpallial Boundary (PSB)	n=3	*Htr3a*-GFP$^+$	n=999	MEIS2/PROX1	Figure 4a
	PSB	n=3	*Htr3a*-GFP$^+$/MEIS2$^+$	n=263	SP8/COUPTFII	Supplementary Figure 7a, b
E18	PSB	n=3	*Htr3a*-GFP$^+$	n=909	MEIS2/PROX1	Figure 4b
			Htr3a-GFP$^+$/MEIS2$^+$	n=468	ER81	Supplementary Figure 4c
				n=369	SP8/COUPTFII	Supplementary Figure 7d, e
	Subventricular Zone (SVZ)	n=3	*Htr3a*-GFP$^+$	n=1219	MEIS2/PROX1	Figure 4b
			Htr3a-GFP$^+$/MEIS2$^+$	n=228	MEIS2/ER81	Supplementary Figure 7c
				n=175	SP8/COUPTFII	Supplementary Figure 7d, e
	Intermediate Zone (IZ)	n=3	*Htr3a*-GFP$^+$	n=981	MEIS2/PROX1	Figure 4b
			Htr3a-GFP$^+$/MEIS2$^+$	n=144	MEIS2/ER81	Supplementary Figure 7c
				n=140	SP8/COUPTFII	Supplementary Figure 7d, e
E14 DIV 2	IZ	n=3	CGE-derived *Htr3a*-GFP$^+$	n=156	None	Figure 4e, f, Supplementary Movie 2
			PSB-derived *Htr3a*-GFP$^+$	n=47	None	Figure 4e, f, Supplementary Movie 1
P5	White Matter (WM)	n=3	*Htr3a*-GFP$^+$	n=2567	MEIS2	Figure 2a
				n=2426	MEIS2/PROX1	Figure 2b
				n=1379	MEIS2/ER81	Figure 2c
				n=1344	MEIS2/SP8	Figure 2d
				n=2127	MEIS2/COUPTFII	Figure 2e
	WM BrdU analysis	n=3	*Htr3a*-GFP$^+$/MEIS2$^+$	n=1293	BrdU inj. E14	Figure 4c
				n=1043	BrdU inj. E15	
				n=944	BrdU inj. E16	
				n=965	BrdU inj. E17	
				n=1410	BrdU inj. E18	
	Cortex (CTX)	n=3	*Htr3a*-GFP$^+$	n=4658	MEIS2	Figure 2a
				n=3018	MEIS2/PROX1	Figure 2b
				n=2528	MEIS2/ER81	Figure 2c
				n=1924	MEIS2/SP8	Figure 2d
				n=2436	MEIS2/COUPTFII	Figure 2e
				n=4396	COUPTFII	Figure 5a
	CTX/WM	n=5	*Dlx5/6*- GFP$^+$	n=354	MEIS2	Supplementary Figure 8a
		n=4	*Dlx5/6-Meis2*-*tGFP* /*Dlx5/6*- GFP$^+$	n=98	MEIS2	Supplementary Figure 8a
		n=5	*Dlx5/6*- GFP$^+$	n=354	None	Supplementary Figure 8b, c
		n=7	*Dlx5/6-Meis2*-*tGFP* /*Dlx5/6*- GFP$^+$	n=199	None	Supplementary Figure 8c
P21	WM	n=3	*Htr3a*-GFP$^+$	n=294	MEIS2	Figure 3a
			Htr3a-GFP$^+$/MEIS2$^+$	n=260	MEIS2/PROX1	Figure 3b
				n=132	MEIS2/COUPTFII	Figure 3b
				n=106	MEIS2/ER81	Figure 3b
				n=70	MEIS2/SP8	Figure 3b
	CTX	n=3	*Htr3a*-GFP$^+$	n=891	MEIS2	Figure 3a
				n=573	COUPTFII/REELIN	Figure 5c
				n=1570	COUPTFII	Figure 5b
